# Material‐Induced Nuclear Deformation Controls Chromatin Architecture in Adipose Stem Cells

**DOI:** 10.1002/advs.202514458

**Published:** 2026-01-30

**Authors:** Carlo F. Natale, Luca Messina, Valeria Panzetta, Stefania Saporito, Costantino Menna, Maurizio Ventre, Paolo A. Netti

**Affiliations:** ^1^ Center For Advanced Biomaterials for Health Care Istituto Italiano di Tecnologia Naples Italy; ^2^ Interdisciplinary Research Centre on Biomaterials University of Naples Federico II Naples Italy; ^3^ Department of Chemical, Materials and Industrial Production Engineering University of Naples Federico II Naples Italy; ^4^ Department of Structures for Engineering and Architecture (DIST) University of Naples Federico II Naples Italy

**Keywords:** actin cytoskeleton, cell mechanics, heterochromatin, nucleus deformation

## Abstract

The 3D organization of the nucleus is crucial for maintaining cellular homeostasis and function, and it is dynamically regulated by both internal and external forces. Here, we investigate how material‐induced cell deformation—generated by engineered micropatterned substrates—influences nuclear morphology and chromatin condensation in adipose‐derived stem cells (ASCs). Using a multiscale approach that integrates mechanical modeling, atomic force microscopy (AFM), confocal imaging, and high‐resolution analysis, we show that the surface micropatterning modulates intracellular force distributions, which in turn reshape the nuclear envelope and alter chromatin organization. Finite Element simulations reveal that distinct deformation profiles lead to region‐specific mechanical stress across the nuclear envelope. These mechanical cues correlate with local chromatin decondensation, as demonstrated by 3D chromatin reconstructions and quantitative morphometric analyses. Our findings demonstrate that cell mechanical perturbations imposed by single‐cell micropatterning can shape chromatin architecture and chromosome inter‐distances. This opens new avenues for understanding mechanogenomic regulation and designing biomaterials that harness physical cues to control cell behavior.

## Graphical abstract

Tuning cell and cytoskeleton mechanics modulated nuclear shape and heterochromatin organization in ASCs. Distinct cytoskeletal architectures induced nuclear morphologies from oblate to prolate ellipsoids. Large elongated cells with a structured actin cap exhibited high nuclear strain, driving nuclear envelope deformation and heterochromatin decondensation. These findings revealed how substrate‐imposed forces reshaped nuclear architecture and promoted chromatin relaxation through mechanical regulation.

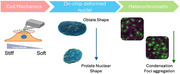

## 1 Introduction

Within the cell nucleus, DNA is organized into chromatin, exhibiting different levels of supramolecular structures. Double‐strand DNA is intricately wound around specialized protein cores known as histones. The interactions between histones and DNA play a pivotal role in modifying the 3D structure of chromatin fibers, influencing the conformation and accessibility of gene regulatory regions [1, 2]. Chromatin can be found in two main configurations: (i) chromatin fibers compacted into a condensed conformation (heterochromatin), impeding the access of transcription factors to DNA and leading to gene repression [3, 4]; (ii) decondensed chromatin fibers (euchromatin), allowing the transcription machinery to access DNA filaments, hence initiating transcription [5]. Therefore, the establishment and maintenance of specific gene‐expression programs heavily rely on the chromatin folding landscape [6].

The 3D organization of chromatin fibers is a dynamic process influenced by various cellular mechanisms, including differentiation, development, and responses to environmental cues. Intracellular stresses and strains were shown to influence the 3D spatial arrangement and packing of chromatin, leading to dynamic regulation of its structure, ultimately resulting in an upregulation of specific genes [7]. Nevertheless, in response to mechanical constraints, cells undergo chromatin condensation, thereby adopting a transcriptionally repressed state [8, 9]. These mechanisms are at the foundation of mechanotransduction, by which cells sense and react to mechanical stimuli, regulating gene transcription [10]. The magnitude, orientation, and spatial distribution of mechanical forces, along with dynamic transmission from the cytoplasm to individual chromatin fibers, are expected to play crucial roles in shaping chromatin architecture [11]. Here, actin stress fibers play an active role in the deformation of chromatin. Due to their physical connection with the nuclear envelope through LINC complexes [12] actin stress fibers are capable of transferring stress across it.

The cell nucleus can be deformed by either externally applied or inborne cytoskeleton generated forces [13, 14]. Examples of techniques involving externally applied forces include atomic force microscopy (AFM) [15], biaxial stretching devices [16], magnetic tweezers [17], and micropipette nuclear aspiration [18, 19]. Here, the modification of heterochromatin condensation state is closely related to the type, duration, and magnitude of the applied mechanical stress [20, 21]. Another approach consists of modulating cytoskeleton mechanics by using engineered culturing platforms. Along these lines, by an opportune presentation of arrays of biochemical/biophysical cues at the cell‐material interface, we have proposed a novel class of cell mechano‐modulating chips that enable the coordinated regulation of the spatial organization of actin stress fibers above the cell nucleus through the control of focal adhesions (FAs) growth and maturation [22, 23, 24]. FAs are integrin‐based complexes that link the extracellular matrix to the cytoskeleton, allowing cells to sense and respond to mechanical cues [25]. Submicrometric confinement of FAs and micrometric confinement of cell shape were shown to influence cytoskeletal mechanics along with actin stress fiber assembly. These structures, in turn, proved to be highly effective in deforming the nuclei [23, 24, 26]. However, the specific influence of nuclear mechanical stimulation on the structural changes of chromatin remains elusive. Unravelling such mechanisms could potentially open avenues for a novel paradigm in cell programming. In principle, the mechanical stimulation of the cell nucleus might activate the expression of genes enclosed within typically inaccessible regions of the genome, thereby tuning the repertoire of genes expressed by cellular machinery.

In this study, we aim to investigate how nuclear deformation influences the spatial reorganization of chromatin, with a specific focus on identifying the spatial positioning of heterochromatin decondensation occurrences in Adipose Stem Cells (ASCs). By means of single‐cell micropatterning, we manipulated cell shape and mechanics to generate four cases of nuclear deformation. In particular, by constraining cell adhesion and shape, we forced the ASCs' cytoskeleton to adopt defined architectures, thereby modulating axial and lateral forces acting on the nuclear envelope and inducing controlled nuclear morphologies ranging from prolate to oblate shapes. Within this framework, we adopted a multiscale experimental–computational strategy in which AFM measurements were employed to experimentally validate micropattern‐induced changes in perinuclear mechanics. Together with a thorough morphological characterization of nuclei, advanced 3D Finite Element Modeling (3D FEM) was used to resolve how these global mechanical perturbations propagate into local nuclear stress distributions and volume variations. This integrated approach enabled a quantitative characterization of the forces acting on the nuclear envelope and their relationship with nuclear deformation in cells cultured on patterned substrates. Finally, leveraging these mechanically defined conditions, we measured both the extension and positioning of chromosomal territories (CTs) and assessed the spatial organization of heterochromatin in response to the different nuclear deformation states.

## 2 Results

### 2.1 Tuning ASCs Cytoskeleton Mechanics by Means of Surface Micropatterning

**FIGURE 1:**
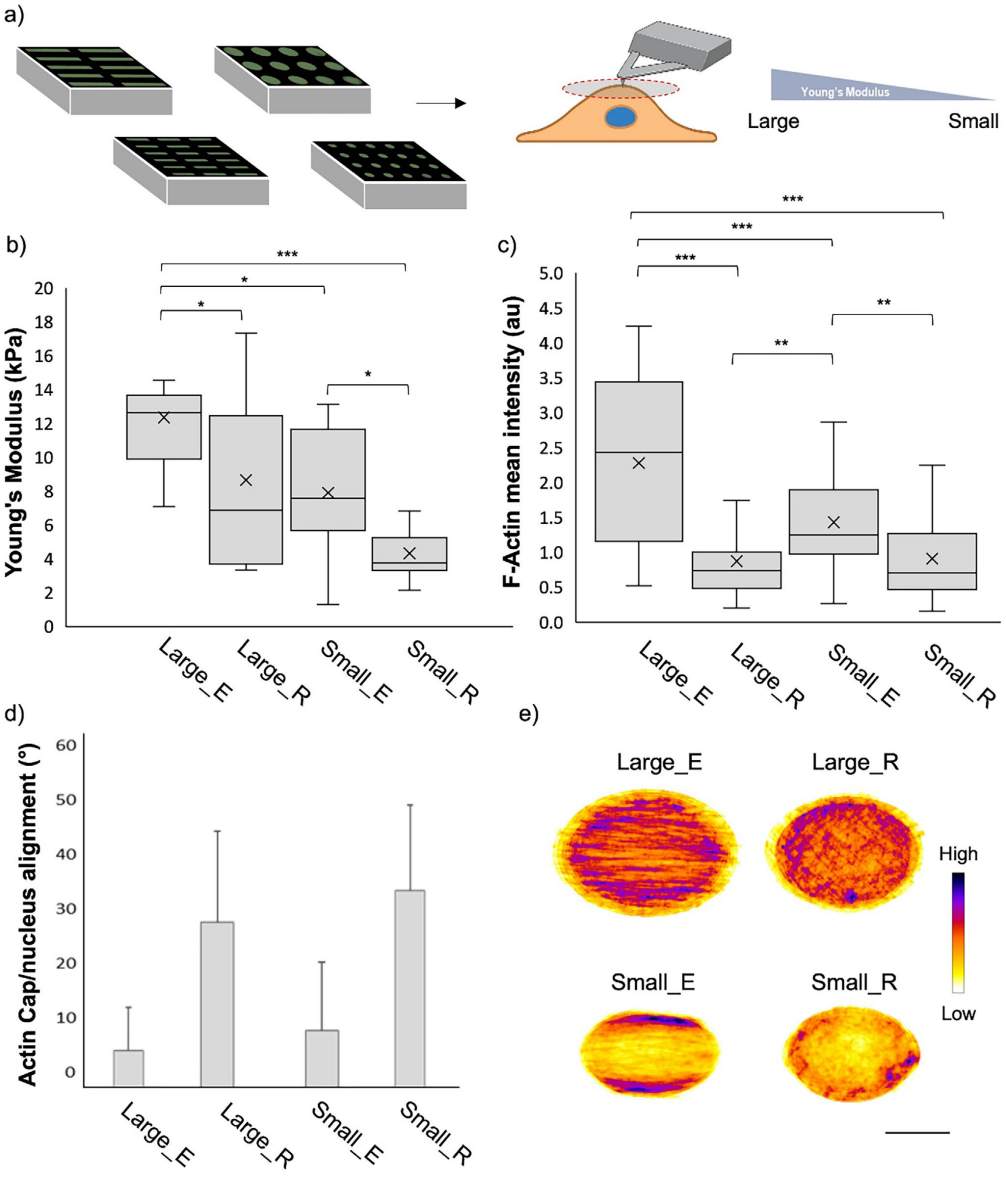
Modulation of cell mechanics and cytoskeleton organization. (a) Schematic representation of AFM measurements. (b) Young's modulus of the perinuclear region of ASCs cultured on micropatterned culturing surfaces. n = 12 cells from two independent experiments for each case (linear mixed effect model test was performed, ^*^ *p*< 0.05; ^**^ *p*< 0.001; ^***^ *p*< 0.0001). (c) F‐actin intensity mean and (d) Actin Cap/ Nuclear coalignment of ASCs cultured on micropatterned surfaces. n = 40 cells from three independent experiments for each case. Kruskal–Wallis, Post‐Hoc Dunn's test (^*^ *p*< 0.05; ^**^ *p*< 0.001; ^***^ *p*< 0.0001). (e) Color‐coded distribution maps of the actin cytoskeleton in the perinuclear area for all experimental conditions. n = 10 cells per condition. Scale Bar is 10 µm.

Morphological changes in cells are accompanied by structural modifications of the actin cytoskeleton and, consequently, cell mechanics. In particular, the cell Young's modulus shows a positive correlation with the cell spreading area, which is associated with the formation of stress fibers in well‐spread cells [27]. To drive cells to acquire different mechanical characteristics, we designed culturing chips featuring fibronectin‐coated islands shaped as circles and rectangles with varying sizes (Figure 1a; Figure S1a). This design aimed to force ASCs to adopt either an elongated (E) or round (R) morphology. Micropatterned islands with surface area extensions of 5000 µm^2^ (Large_E, Large_R) and 1600 µm^2^ (Small_E, Small_R) were specifically engineered to modulate the spreading of ASCs either under or over the adhesive surface area typically occupied by the same cells when cultured on unpatterned fibronectin‐coated surfaces (Figure S1b). To demonstrate the substrate capability to modulate ASCs' mechanics, we evaluated Young's Modulus of living ASCs after 24 h of culture by means of AFM (Figure 1b). In particular, we performed the indentation on the top of cell nuclei, a procedure commonly used to evaluate the global cell mechanics [28]. We found that cells cultured on large adhesive islands (i.e, Large_E, Large_R), possessed stiffer perinuclear moduli compared to those cultured on small adhesive islands (i.e, Small_E, Small_R). However, modification of cellular shape led to increased stiffness in the apical nuclear regions. Specifically, Large_E ASCs maintained a stiffer apical nuclear region compared to their Large_R counterparts. Similarly, Small_E exhibited significantly higher moduli compared to their counterparts with a round morphology (Small_R). Then, we validated the substrate's capability to modulate ASCs mechanical state, evaluating FAs assembly, which is known to correlate with an overall increase of tension acting within the cell [29]. Therefore, we analyzed confocal images of paxillin‐labeled ASCs. FAs features, such as elongation and orientation, were here employed as indicators of the cell's mechanical state and marked differences between experimental groups were observed (Figure S2a). More specifically, Large_E ASCs formed FAs that were significantly longer and with a higher aspect ratio with respect to FAs in Large_R cells (Figure S2b,c). Similarly, Small_E ASCs could form longer FAs with respect to those observed in Small_R ASCs. In addition, we observed that elongation promoted the formation of FAs oriented with respect to cellular elongation axis (average angles of 10° ± 0.7° and 11° ± 0.9°, respectively; *p*< 0.05; (Figure S2d), while Large_R and Small_R ASCs formed FAs that were randomly oriented around cell body (average angle of 40.6° ± 0.7°). Overall, these analyses suggest that ASCs exhibit an increased stiffness when adopting an elongated morphology compared to their round counterparts, with Large_E displaying the highest stiffness among the experimental groups.

As the Young's modulus of the perinuclear region measured by AFM is affected by different cell compartments, we assessed how distinct actin cytoskeleton assemblies contribute to defining the mechanical characteristics of the apical nuclear region. We acquired a confocal z‐stack of ASCs cytoskeleton (actin) and nuclei to study the organization of actin stress fibers around the nuclear region, known as actin‐cap fibers. Large_E ASCs displayed prominent, thicker actin stress fibers that crossed the entire cellular body, accompanied by well‐developed actin cap fibers running along the nuclear polarization axis (Figure S3a). In stark contrast with this observation, Large_R ASCs exhibited an actin cytoskeleton characterized by transverse arcs running parallel along the geometrical boundaries of the micropattern, along with the presence of dorsal stress fibers arranged radially across the entire cellular body. Also, fibers forming an actin‐cap were present (Figure S3b). On small adhesive islands, Small_E ASCs possessed an actin cytoskeleton mostly constituted by actin stress fibers packed by the lateral long edges of fibronectin coated island, and fibers forming actin‐cap were also observed (Figure S3c). Conversely, Small_R ASCs showed small patches of whiskers on the top of the apical nuclear surface (Figure S3d). To further characterize the different cytoskeleton assemblies forming above the nuclear envelope, we quantified the F‐actin mean fluorescence intensity of the ASC actin‐cap, utilizing a 3D confocal section obtained from TRITC‐phalloidin staining (Figure 1c). In agreement with cell stiffness measurements, Large_E ASCs exhibited the highest content of F‐actin compared to other experimental groups. The elongation of ASCs resulted in the assembly of thick fluorescent bundles of parallel fibers running along the entire nuclear surface. Cellular elongation promoted the assembly of a thicker actin cap even when ASCs were cultured on small adhesive islands. Indeed, Small_E ASCs displayed a higher F‐actin mean value compared to those calculated for Small_R ASCs. However, the content of F‐actin for Small_E ASCs was lower compared to Large_E cells. To measure the distribution of orientation of actin cytoskeleton fibers above the nuclear envelope, we measured the orientation of cytoskeleton fibers relative to the nuclear polarization axis. We observed that elongated cells exhibited actin‐cap fibers that were highly aligned with the nuclear polarization axis, whereas such alignment was not observed in round cells. (Figure 1d). Maps in Figure 1e show the preferential distribution of actin fibers in the perinuclear region. Despite the strong co‐alignment with nuclear orientation in elongated cells, the organization of actin‐cap stress fibers above the nuclear envelope differed between Large_E and Small_E ASCs. Indeed, the distribution map for Large_E revealed that actin fibers were preferentially positioned above the entire cell nuclear surface. It is worth noting that a faint donut‐like pattern, more evident in Large_E cells, can be observed at the map periphery. This feature arises from minor edge effects due to differences in nuclear size across cells (see Materials and Methods section). In contrast, the distribution maps for Small_E ASCs showed that actin‐cap bundles of fibers were predominantly localized on the lateral side of the cell nucleus. Then, the analysis of the area above the cell nucleus occupied by actin cap fibers, as well as their average number, was performed on phalloidin z‐stack images for experimental conditions in which the actin cap was observed. (i.e. Large_E, Large_R and Small_E). Our results showed that actin cap fibers occupied 60 %, 47 %, and 53 % of the apical region of the cell nucleus in Large_E, Large_R, and Small_E, respectively (Figure S3e). We observed a decrease in the number of actin stress fibers from Large_E (10) to Large_R (9) and Small_E (5) (Figure S3f). Overall, our data showed that in each experimental condition, ASCs possessed peculiar mechanical characteristics, where an increase in adhesion state induced an overall increase in stiffness of the apical perinuclear regions.

### 2.2 Tuning ASC Mechanics Induced for Specific Cases of Nuclear Deformation

**FIGURE 2:**
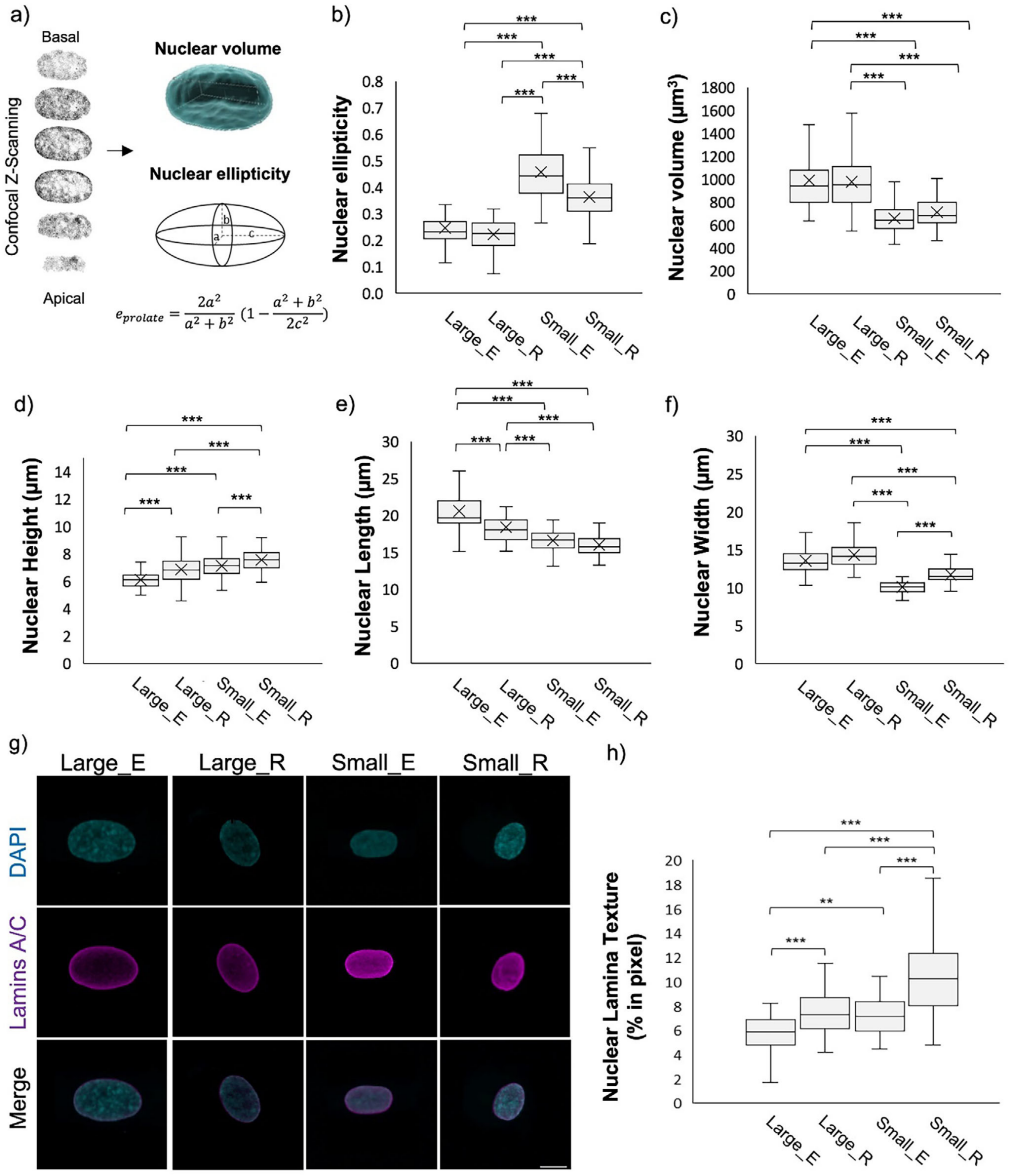
Distinct arrangements of the actin cytoskeleton induce specific nuclear deformations. (a) Imaris‐generated surface from confocal z‐scanning of the ASCs DAPI labeled nuclei, where the 3D surface (blue) encloses the entire contents of the nucleus. Box Plots of (b) cell nucleus ellipticity, (c) volume, (d) height, (e) length, and (f) width of ASCs cultured on micropatterned surfaces for 24 h. n = 60 cells from four independent experiments for each case. Kruskal–Wallis, Post‐Hoc Dunn's test (^*^ *p*< 0.05; ^**^ *p*< 0.001; ^***^ *p*< 0.0001). (g) Representative immunofluorescence images of ASCs nuclei immunostained for Lamin A/C for all tested conditions. Scale bar is 10 µm. (h) Box Plot showing Lamin A/C texture calculated as pixels in texture/total nuclear pixels. n = 40 nuclei from three independent experiments for each case were calculated. Kruskal–Wallis, Post‐Hoc Dunn's test (^*^ *p*< 0.05; ^**^ *p*< 0.001; ^***^ *p*< 0.0001).

We then investigated whether differently arranged forces acting on the nuclear envelope could induce specific deformations in the cell nucleus. 3D renderings of the nuclear surface were obtained from confocal z‐stacks of DAPI‐labeled nuclei for ASCs cultured on the four different substrates. Nuclear morphological changes were first registered as a variation of nuclear 3D ellipticity, which provided information on the deviation from the spherical configuration that we assumed to be the reference configuration (Figure 2a). Smaller values of ellipticity indicated a more oblate shape of the nucleus, whereas higher ones referred to a more prolate cigar‐shaped ellipsoid. We noted that Large ASCs had nuclei more oblate, indicating greater flattening along nuclear height. In contrast, Small ASC exhibited nuclei with a more prolate ellipsoidal shape (Figure 2b). However, Small_E ASCs exhibited an even more prolate shape compared to their Small_R ASC counterparts, while no statistical difference between Large_E and Large_R ASCs was observed. The values of the nuclear volume indicated that nuclei of Large ASCs attained significantly larger volumes (Large_E = 990.26 ± 258.00 µm^3^, Large_R = 977.29 ± 257.22 µm^3^) with respect to those calculated for cells cultured on small adhesive islands (Small_E = 650.33 ± 117.13 µm^3^; Small_R = 711.59 ± 130.39 µm^3^ (Figure 2c).

Afterward, we estimated other relevant nuclear shape descriptors (height, length, and width) for all tested conditions. We noted that a gradual decrease in apical nuclear stiffness correlated with an increase in ASC nuclear height. Nuclei exhibited greater height when ASCs spread on small adhesive islands compared to their larger counterparts (Figure 2d). Then, we observed that Large_E possessed longer nuclei with respect to all other tested groups. However, cellular elongation did not elicit such an effect when ASCs were cultured on small adhesive islands, in which case no statistical differences between Small ASCs groups were observed (Figure 2e). Furthermore, the organization of actin stress fibers in Small_E ASCs strongly deformed nuclei, confining their lateral expansion and reducing their width (Figure 2f).

Since the cell cycle could play a role in determining nuclear shape [30], we evaluated the nuclear morphological characteristics of ASCs in the same phase, such as G1. To identify nuclei in the G1 phase, we plotted the histogram of the DNA content of the entire cell population based on DAPI‐stained samples, as described elsewhere [31]. Modifications in the distribution of actin stress fibers are responsible for changes in nuclear morphology in the G1 phase as well. Here, nuclear shape descriptors, along with nuclear volume and ellipticity, exhibited similar trends when comparing ASCs in the G1 phase and cells spanning all cell‐cycle stages, with statistical differences preserved between the observed groups (Figure S4). Our findings confirm that nuclear morphology acquired by ASCs cultured on micropatterned surfaces is independent of the cell cycle. Altogether, the data indicated that varying levels of tension over the nuclear envelope caused the cell nucleus to be deformed accordingly. Large_E ASCs possessed well‐structured actin stress fibers that deformed the cell nucleus: (i) inducing an oblate morphology; (ii) increasing its volume; (iii) reducing its thickness; and (iv) promoting its stretching along the cell polarization axis. As the organization of the nuclear lamina is sensitive to changes in nuclear morphology, we characterized the geometrical features of the lamina by means of immunofluorescence for Lamin A/C (Figure 2g). More specifically, we quantified nuclear lamina texture (corresponding to folds, wrinkles, holes, etc.) in the nuclear cross‐section as the number of pixels in grooves, folds, holes, and wrinkles for all tested conditions. Higher values indicate increased wrinkling of the nuclear membrane. The results revealed a negative correlation between Lamins A/C texture and cell perinuclear Young's modulus, in agreement with literature data reporting increased lamina texture occurring in nuclei exhibiting a lower tension [32]. In fact, Lamin A/C texture exhibited the smallest value for Large_E ASCs and the largest for Small_R ASCs, suggesting that increased wrinkling is related to lower stresses acting on the perinuclear region (Figure 2h). These findings suggest that nuclear deformation is accompanied by unfolding of the nuclear lamina, consistent with similar observations where Lamins A/C organization is modified during nuclear flattening and cell spreading [32].

### 2.3 Numerical Approach to Macroscopically Simulate Cellular Spreading and Nuclear Deformation

**FIGURE 3:**
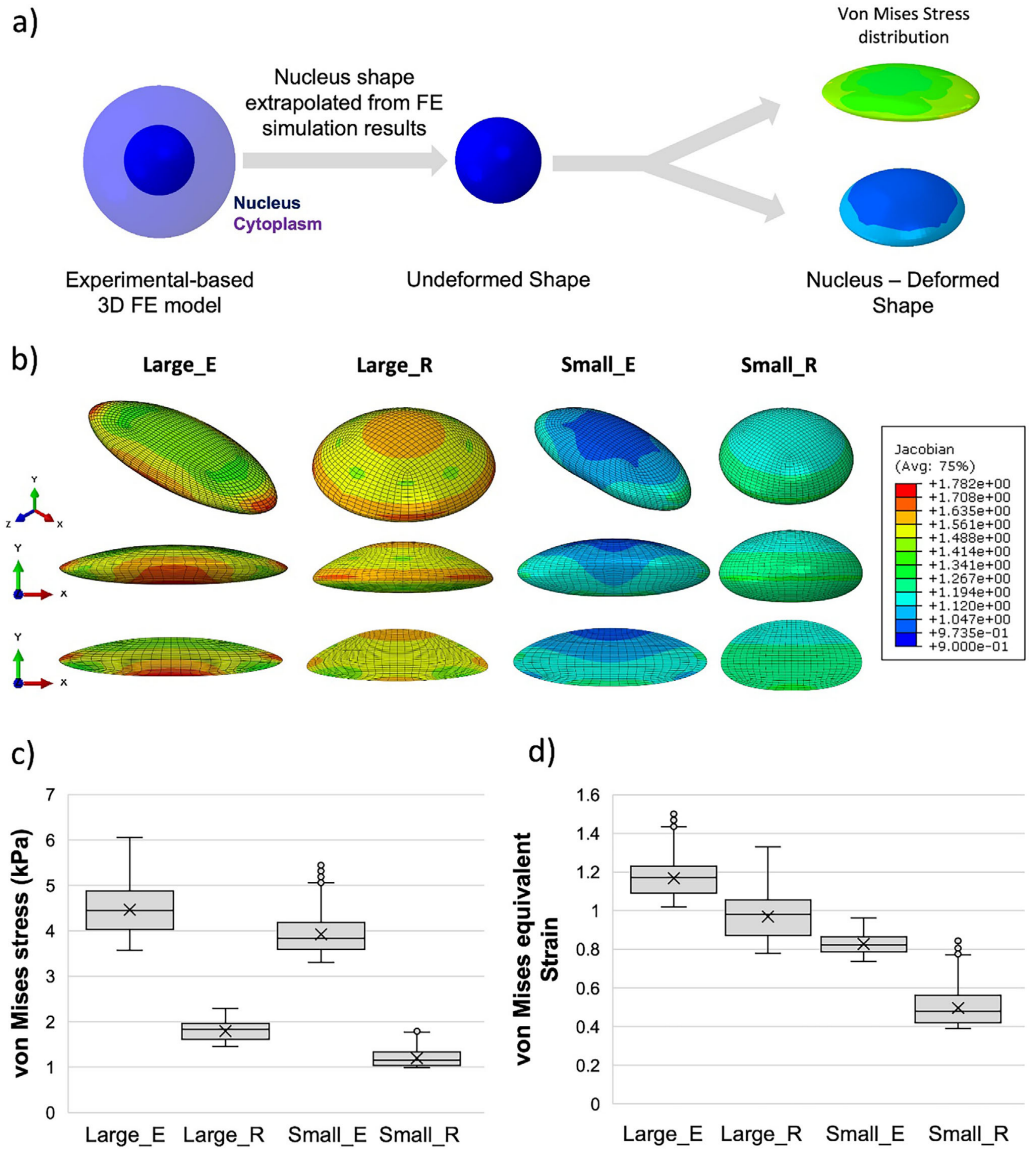
Finite Element simulations reveal distinct nuclear deformation profiles. (a) Schematic representation of experimental‐based 3D FEM. (b) Jacobian distribution of nucleus for all micropatterned substrate configurations. (c) Box plot of von Mises stress (kPa) for all cases. (d) Box plot of von Mises equivalent strain for all cases.

To estimate local stresses and strain of cells subjected to the four different material‐induced mechanical conditions, we developed a finite element (FE) mechanical model (Figure 3a). In particular, we modified a 3D FEM model previously developed [26] that reproduced the cell spreading and nuclear strains on the four different patterned substrates, taking into account cellular and nuclear compressibility. Briefly, to simulate cell spreading, a full 3D FEM model, including cytoplasm, nucleus, and actin cap, was formulated for each configuration herein investigated. The cell spreading was reproduced within the finite deformation hypothesis and through the application of a specific experimentally derived displacement field on the cell outer surface (see Materials and Methods and Supporting Information—The 3D Finite Element Model Approach). Specifically, the kinematic equations of the displacement fields were formulated to reproduce the same cell morphology observed in the experiments after 24 h of culture. In Figure S5, the different displacement fields obtained on the cell surface from 3D FEM simulations are shown for all pattern situations. A good agreement of cell area (length and width) and cell height was observed between experimental and numerical approaches (Figure S6).

To validate the numerical approach, the nuclear volume, nuclear height, length, and width of experiments and simulations were compared (Figure S7). All the quantities evaluated with the 3D FEM approach were in the same order of magnitude with respect to the experimental results, even if a slight underestimation of nuclear volume and nuclear height from simulative outcomes was observed (Figure S7a,b). In particular, the introduction of nuclear compressibility allows the model to capture reasonably well the nuclear volume dilations observed experimentally (Figure S7a). Then, we extracted the Jacobian (i.e., the ratio between the deformed and undeformed element volume) for each element of the finite element mesh and used it to generate the contour map reported in Figure 3b. Local deformations are strongly region‐dependent and pattern‐specific, with the largest deviations occurring in nuclear regions aligned with the principal deformation axes imposed by the micropattern geometry (especially for larger patterns, Figure S8a). Finally, the Jacobian profiles for each element were plotted along the major (Figure S8b) and minor (Figure S8c) axes of the deformed nuclei to provide information on the local volumetric changes occurring inside the nuclei. In more detail, starting from the central point on the equatorial plane of the nucleus, those profiles were obtained for each element along the major/minor axis, and due to the symmetry in the x‐y plane, only one‐half of the major/minor axis was considered. We observed the highest increase in the Jacobian for ASC cultivated on large adhesive islands. Increases were particularly pronounced in the peripheral regions. Small ASC displayed a lower Jacobian increase with respect to Large ASC. However, also in this case, the largest volumes were generally located at the cell periphery (Figure S8b,c). Then, we calculated the von Mises stress and von Mises equivalent strain on the nuclear surface across the different configurations (Figure 3c,d). As shown in Figure 3c, comparable von Mises stress distribution was quantified between round (Small_R, Large_R) and elongated (Small_E, Large_E) cases, and larger adhesion areas result in higher stress values. However, as shown in Figure 3d, von Mises equivalent strain is deeply correlated with ASC stiffness. Particularly, Large_E ASCs develop larger stains with respect to other experimental groups. However, Small_E ASCs possessed a higher strain profile with respect to the round counterpart (ie, Small_R). The 3D FEM allowed us to depict the non‐homogeneous stress and strain states within the nuclei. The model predicts that position‐dependent and finite volume changes occur, events that potentially impact the structural organization of chromatin.

### 2.4 Nuclear Deformation Impacted CTs Organization and Positioning

**FIGURE 4:**
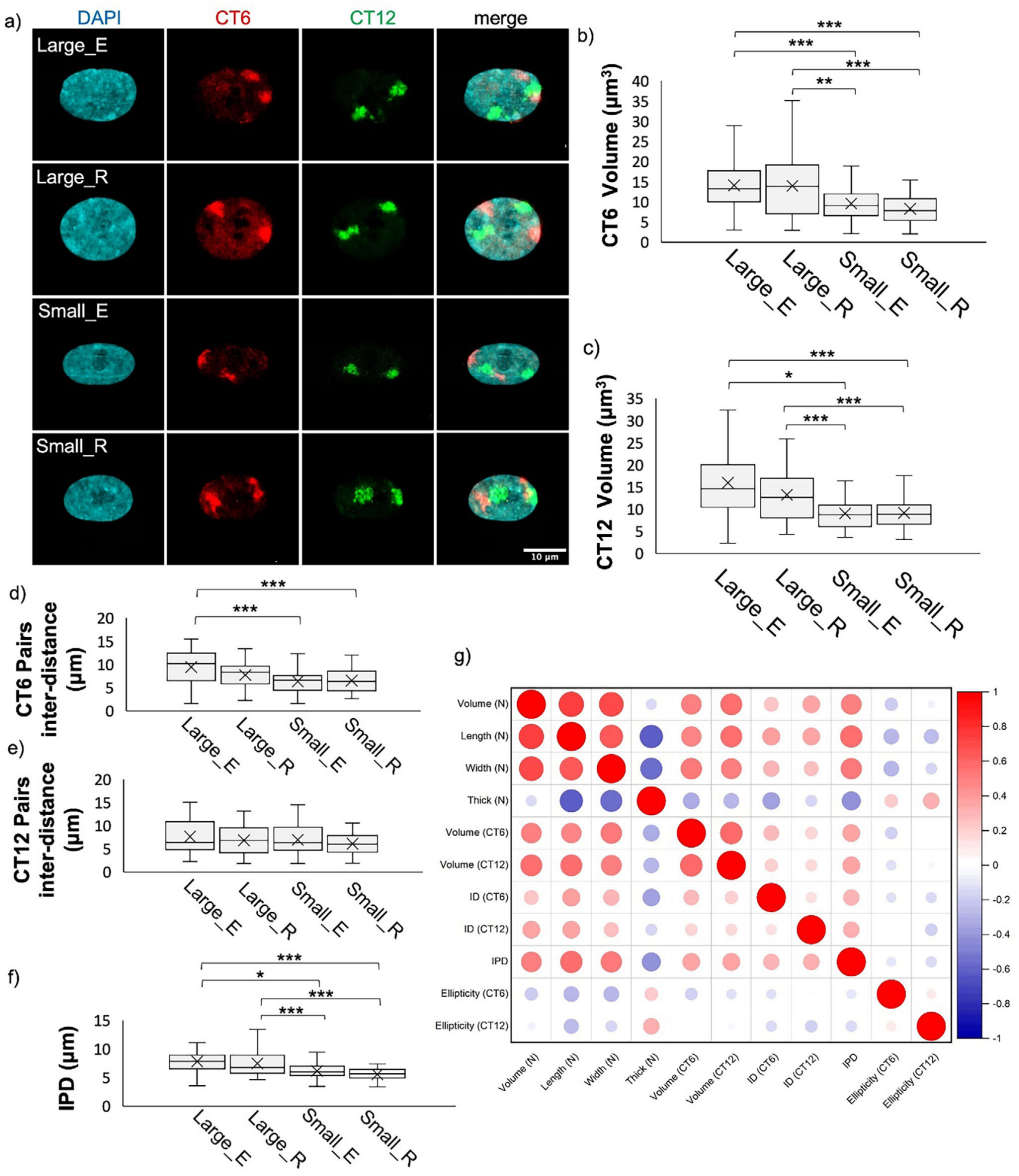
Induced‐nuclear deformations impact CTs organization and positioning. (a) Representative images showing Chromosome Painting of specific CTs (CT6‐red and CT12‐green) of ASCs for all tested conditions. Scale Bar is 10 µm. Quantification of (b,c) chromosome volume, (d,e) pair inter‐distances and (f) inter‐physical distance (ID) for CT6 and CT12, respectively. n = 40 cells for each condition were calculated. Kruskal–Wallis, Post‐Hoc Dunn's test (^*^ *p*< 0.05; ^**^ *p*< 0.001; ^***^ *p*< 0.0001). (g) Heatmap of Pearson correlation coefficient for all nuclear and chromosomal territories features.

The cytoskeleton‐induced nuclear shape changes are likely to affect the 3D organization of nuclear matter. Here, we aim to evaluate the impact of nuclear shape deformation on the organization of CTs. We used 3D‐FISH combined with confocal z‐scanning microscopy to assess the 3D spatial organization of two representative CTs (6 and 12), which differ in gene density and, consequently, in their nuclear localization (Figure 4a) [33, 34]. Considering these distinct spatial patterns, we aimed to assess whether nuclear deformation could impact chromosome territories' morphology and positioning independently of their radial organization within the cell nucleus. We observed that nuclear deformation impacted CTs volume, where the volume of CTs 6 and 12 of Large ASCs attained higher values with respect to those calculated for Small ASC (Figure 4b,c). The process for fluorescence hybridization is known to underestimate nuclear volume [35]. To address this issue, we calculated the CT volumes normalized with respect to the nuclear one, and we observed a trend similar to the previous one, where the normalized volumes of CTs 6 and 12 of Small ASCs groups occupied a smaller volume with respect to Large ASCs ones (Figure S9a,b). CTs 6 and 12 showed a nearly constant nuclear volume between them in all tested conditions (Figure S9c). Then, we assessed if modifications of CTs volume were also accompanied by CT shape changes. We observed that changes in the volume of chromosomal territories do not necessarily correspond to changes in their shape. In fact, significant differences were observed only between Large_E and Small_R groups (Figure S9d,e). To evaluate the impact of imposed nuclear shape on CTs displacement, the relative inter‐distance of CT pairs was quantified. Specifically, we extracted the coordinates of the centroids of two pairs of chromosomes, and their interdistance was calculated. We observed that nuclear shape affected CTs 6 and 12 pairs differently. The two‐paired CT6 distance resulted in higher values in the case of the elongated oblate morphology in Large_E ASCs nuclei with respect to the prolate ones (Small ASCs) (Figure 4d). On the other side, we did not find any statistical difference for the CT12 pairs distance (Figure 4e). Even when homologous CTs maintain a conserved relative positioning, their spatial relationships with respect to other CTs could still change as a consequence of nuclear deformation. To address this issue, we measured the inter‐physical distance (IPD) between CTs 6 and 12 [36]. Our results suggested that specific deformation experienced by cell nuclei can also impact the distances between different chromosome territories (Figure 4f). In fact, we observed that nuclei with larger volumes, such as those cultured on large adhesive islands, exhibited higher IPD compared to nuclei of ASCs cultured on smaller adhesive islands.

Finally, to further analyze whether nuclear deformation could affect the shape or positioning of chromosome territories, we constructed a correlation matrix of morphological data (Figure 4g). As expected, a correlation between nuclear morphological features (i.e., nuclear volume, length, width, and thickness) was evident. Moreover, we found a positive correlation between nuclear and CT volumes and between nuclear morphological features and chromosomal volumes. However, our results suggest that the morphological features of nuclei influence the volumetric properties of CTs, but exert a limited impact on CT morphology, aside from a slight oblation likely due to reduced nuclear height. Even if we were not able to identify any significant correlation between chromosome‐pairs interdistance and nuclear features, we found a positive correlation between the inter‐physical distance between CTs 6 and 12 and nuclear features. The IPD appeared to be influenced by nuclear dimensions, specifically the width and length, indicating a spatial reorganization of chromosomal territories on the x‐y plane. These data suggest that chromosomes rearrange within the nucleus in response to deformation but maintain relative distances between homologous pairs stable, indicative of a non‐homogeneous deformation. Overall, our data showed that the nucleus morphology induced by different mechanical conditions could modify the organization of representative CTs within ASCs nuclei, suggesting a potential modification of nuclear matter organization, in a position‐dependent manner.

### 2.5 Nuclear Deformation Impacted Chromatin Compaction States

**FIGURE 5:**
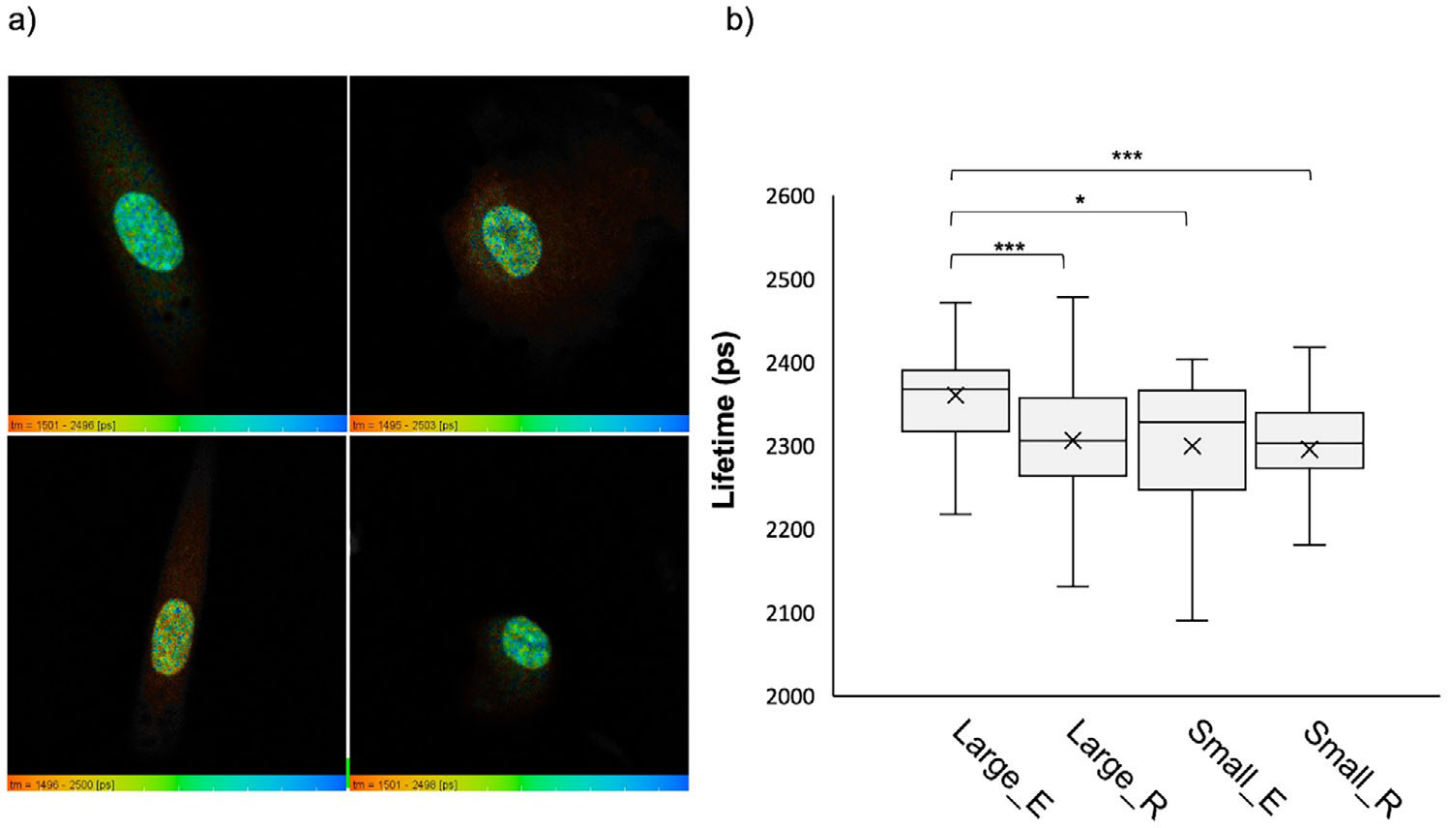
Assessment of chromatin condensation state by means of FLIM microscopy. (a) Representative color‐coded lifetime images (scale ranging from 1497 to 2503 ps) of ASCs nuclei for all tested conditions. (b) Quantification of relative Hoechst 34580 lifetime changes observed for ASCs cultured on micropatterned surface. n = 37 nuclei per condition obtained from three independent experiments were calculated. Kruskal‐Wallis, Post‐Hoc Dunn's test (^*^ *p*< 0.05; ^**^ *p*< 0.001; ^***^ *p*< 0.0001).

Here, we investigate the impact of cytoskeletal‐induced nuclear shape on the supramolecular organization of chromatin. First, we quantified chromatin condensation in fixed cells using Fluorescence Lifetime Imaging (FLIM) of Hoechst‐stained samples. Condensed chromatin is characterized by a low mean fluorescence lifetime, while decondensed chromatin possesses a high mean fluorescence lifetime [37]. Digital maps of the local chromatin organization (i.e., condensation degree) are shown in Figure 5a, in which hot colors are associated with low fluorescence lifetimes and cold colors are related to high fluorescence lifetimes. FLIM data were analyzed using the phasor plot approach to map Hoechst fluorescence lifetimes and cluster nuclear pixels within defined regions of the phasor plot, which limited our analysis to pixels corresponding to the nuclear region (Figure S10). We observed that the average mean fluorescence lifetime of Large_E ASC nuclei was higher with respect to all other tested conditions. This suggests that Large_E ASCs exhibited a lower extent of chromatin condensation. (Figure 5b).

**FIGURE 6:**
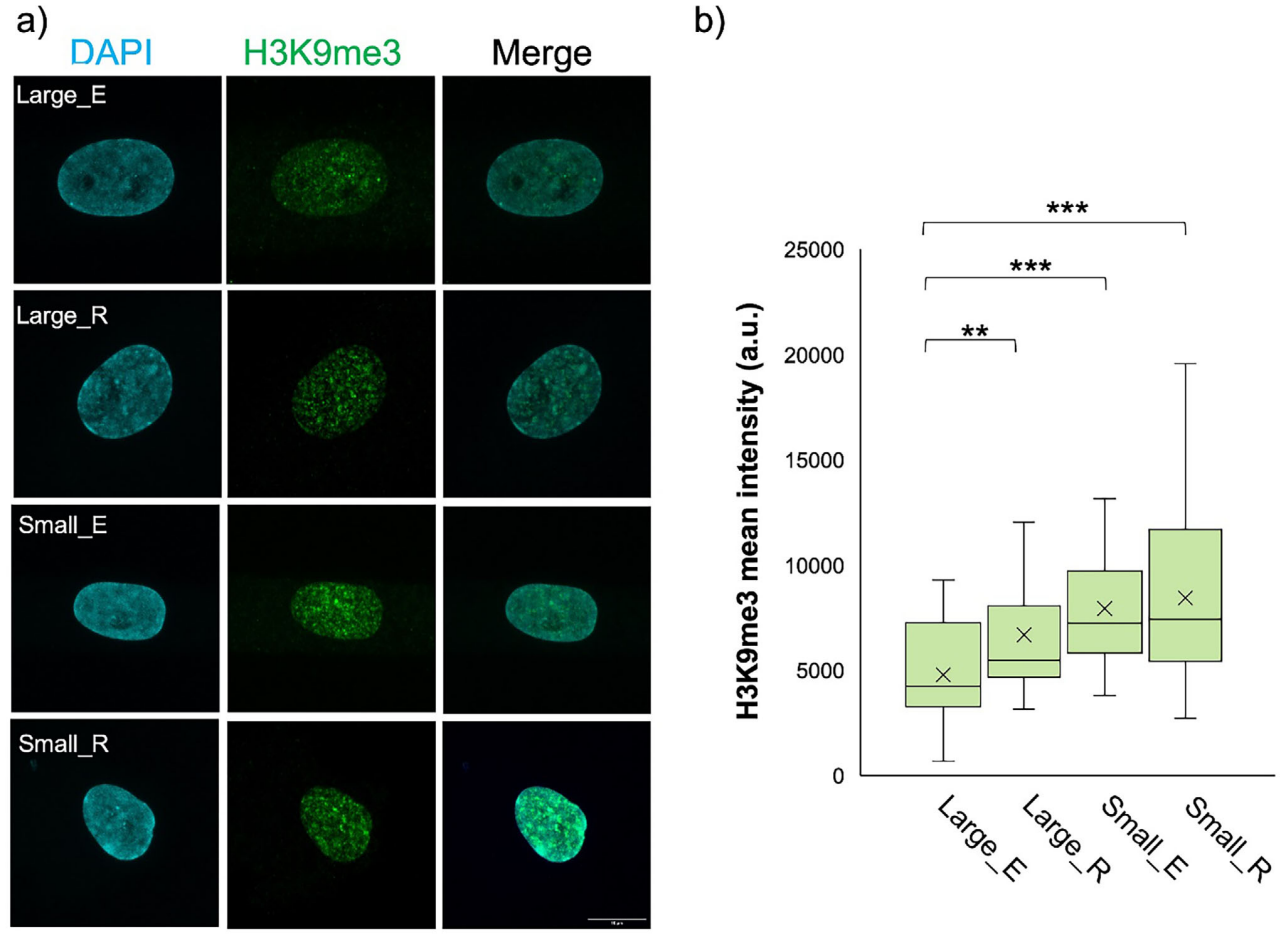
Induced‐nuclear deformations influence H3K9me3 condensation state. (a) Representative immunofluorescence confocal images of nuclei (cyan) and H3K9me3 (green) of ASCs cultured on micropatterned surfaces. The scale bar is 10 µm. (b) Quantification of H3K9me3 intensity levels per nuclear volume within ASCs nuclei for all tested conditions. n = 40 nuclei for each condition from three independent experiments. Kruskal–Wallis, Post‐Hoc Dunn's test (^*^ *p*< 0.05; ^**^ *p*< 0.001; ^***^ *p*< 0.0001).

Second, we measured fluorescence intensity of z‐stacks of H3K9me3‐marked nuclei (Figure 6a). H3K9me3 is an epigenetic modification that is a well‐established marker of constitutive heterochromatin. 3D surfaces enclosing the entire nucleus were used to determine H3K9me3 pixel intensities. We noted that Large_E ASCs exhibited lower levels of H3K9me3 compared to their Large_R ASCs counterparts, suggesting a reduced state of condensation in constitutive heterochromatin. However, in Small ASCs, the mean values increased even further compared to Large_E ASCs, indicating a widespread condensation of constitutive heterochromatin (Figure 6b). Furthermore, we normalized the H3K9me3 mean intensity to the DAPI signal to account for any variations in staining permeability across different cells. As shown in Figure S11a, this approach did not alter the observed trend. However, we noticed that DAPI normalization shifts the median H3K9me3 values of Small_R closer to Small_E, without affecting the statistical significance between groups. Then, we calculated the volume occupied by H3K9me3 within the nuclear volume of ASCs nuclei, and we observed a significant increase in H3K9me3 volume when comparing Large ASCs with respect to Small counterparts (Figure S11b,c). These results suggest that the increased strains experienced by nuclei of Large_E ASCs retained constitutive heterochromatin in a more decondensed state compared with all other tested conditions.

**FIGURE 7:**
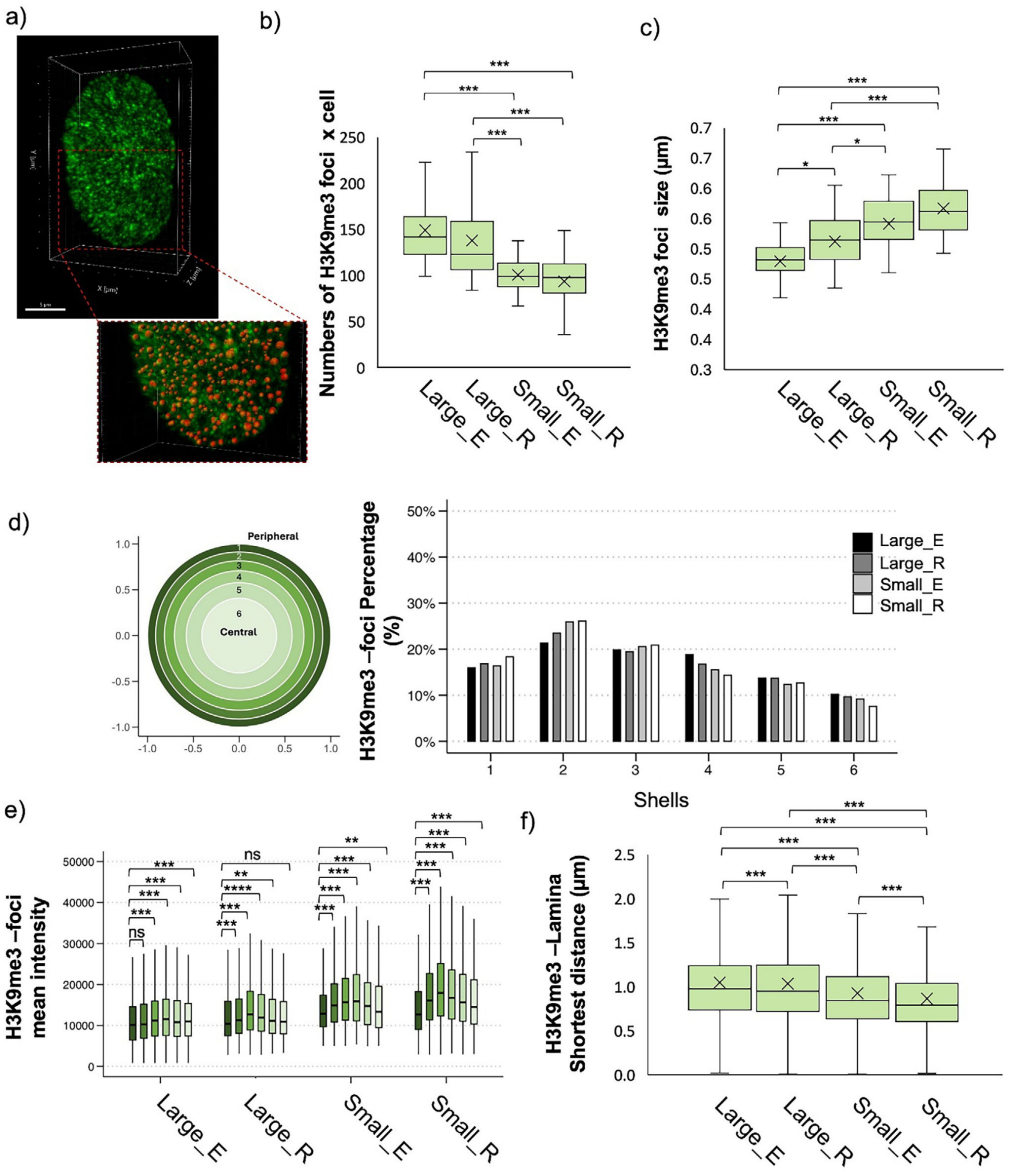
Induced‐nuclear deformations influence H3K9me3 foci spatial organization. (a) Representative 3D individuation of H3K9me3 foci within the cell nucleus. Quantification of the number (b) and the size (c) of H3K9me3 foci for each tested condition. (d) Evaluation of H3K9me3 foci spatial distribution within the cell nucleus, classifying heterochromatin foci into six concentric 3D shells and calculating the percentage of these foci within each one. (e) Evaluation of the foci fluorescence mean intensity within each concentric shell for all tested conditions. (f) Quantification of the distance from the lamina of H3K9me3 domains for each tested condition. n = 40 nuclei for each condition from three independent experiments. Kruskal–Wallis, Post‐Hoc Dunn's test (^*^ *p*< 0.05; ^**^ *p*< 0.001; ^***^ *p*< 0.0001).

Additionally, we assessed the spatial distribution and the size of heterochromatin foci within the nucleus (Figure 7a). First, we extracted the 3D coordinates of the H3K9me3 foci centroids, and we noticed a decreasing trend of the number of H3K9me3 foci from Large to Small ASCs, with significant differences between the experimental groups (Figure 7b). Specifically, Large ASCs exhibited a higher number of heterochromatin foci, but they also displayed a lower degree of chromatin condensation compared to Small ASCs. Then, we calculated the changes in interdistances between heterochromatin foci. Higher values of heterochromatin interdistances for Small ASCs compared to Large ones were observed (Figure S11d,e). Finally, we measured the size of H3k9me3 foci by computing spheres of variable diameter around each heterochromatin foci centroid, with sphere size adapting to the local fluorescence contrast (see Supporting Information‐Image analysis section). We observed that Large_E ASCs nuclei exhibiting a lower degree of chromatin condensation possessed smaller foci with respect to all other tested conditions (Figure 7c). Altogether, our findings support the hypothesis that nuclei undergoing lower deformations exhibit fewer and larger condensed heterochromatin foci, whereas nuclei experiencing higher mechanical stress harbor a larger number of small and less condensed heterochromatin domains.

To evaluate the redistribution of heterochromatin foci relative to the peripheral or central nuclear regions, we calculated H3K9me3 positioning along the radial direction within all tested nuclear deformation cases. In particular, we transformed each ellipsoidal nuclei in unitary sphere, segmented them into 6 concentric spherical shells, and calculated the percentage of heterochromatin foci centroids within each shell. The ellipsoid‐to‐sphere mapping is an affine transformation that preserves heterochromatin centroid relationships. To verify this, we compared the distribution of heterochromatin foci centroid in the native ellipsoid, with ellipsoidal shells, and sphere with spherical shells (Figure S12a,b). The histogram reporting the number of foci assigned to each shell under the two geometries showed identical distributions (Figure S12c). This confirms that the affine mapping preserved spatial relationships and does not introduce bias in the localization of heterochromatin foci. Our results showed that H3K9me3 foci were preferentially located at the nuclear periphery, with Small ASCs showing the highest percentage of the heterochromatin spots within this nuclear region (Figure 7d). Then, we evaluated the mean fluorescence intensity of H3K9me3 foci within each shell. A general increase in fluorescence intensity of heterochromatin foci was observed within the third and fourth shells compared to those calculated for foci in the peripheral one (first shell) for all tested conditions (Figure 7e). However, elongated oblate nuclei in Large_E ASCs did not show any statistical differences within heterochromatin foci populating peripheral nuclear regions (i.e., first and second shells), whereas a significant increase of fluorescence intensity within the same nuclear regions was observed in other experimental groups (Figure 7e).

Then, we measured the distances between H3K9me3 domains and the nuclear lamina. The results indicated that regional heterochromatin distribution was strongly affected by nuclear deformation. A progressive reduction in the distance between H3K9me3 domains and the nuclear lamina was observed from nuclei experiencing lower mechanical strains with respect to those experiencing higher strains (Figure 7f). H3K9me3 heterochromatin domains were found to be closer to the nuclear membrane for Small_R ASCs compared to Large_E. Overall those data indicated that heterochromatin foci aggregate with the lamina membrane around the nuclear periphery when constitutive heterochromatin acquires a more condensed state. In fact, a reduction of stresses generating prolate‐deformed nuclei (i.e. Small ASCs) relocated their more condensed heterochromatin foci toward the nuclear lamina.

**FIGURE 8:**
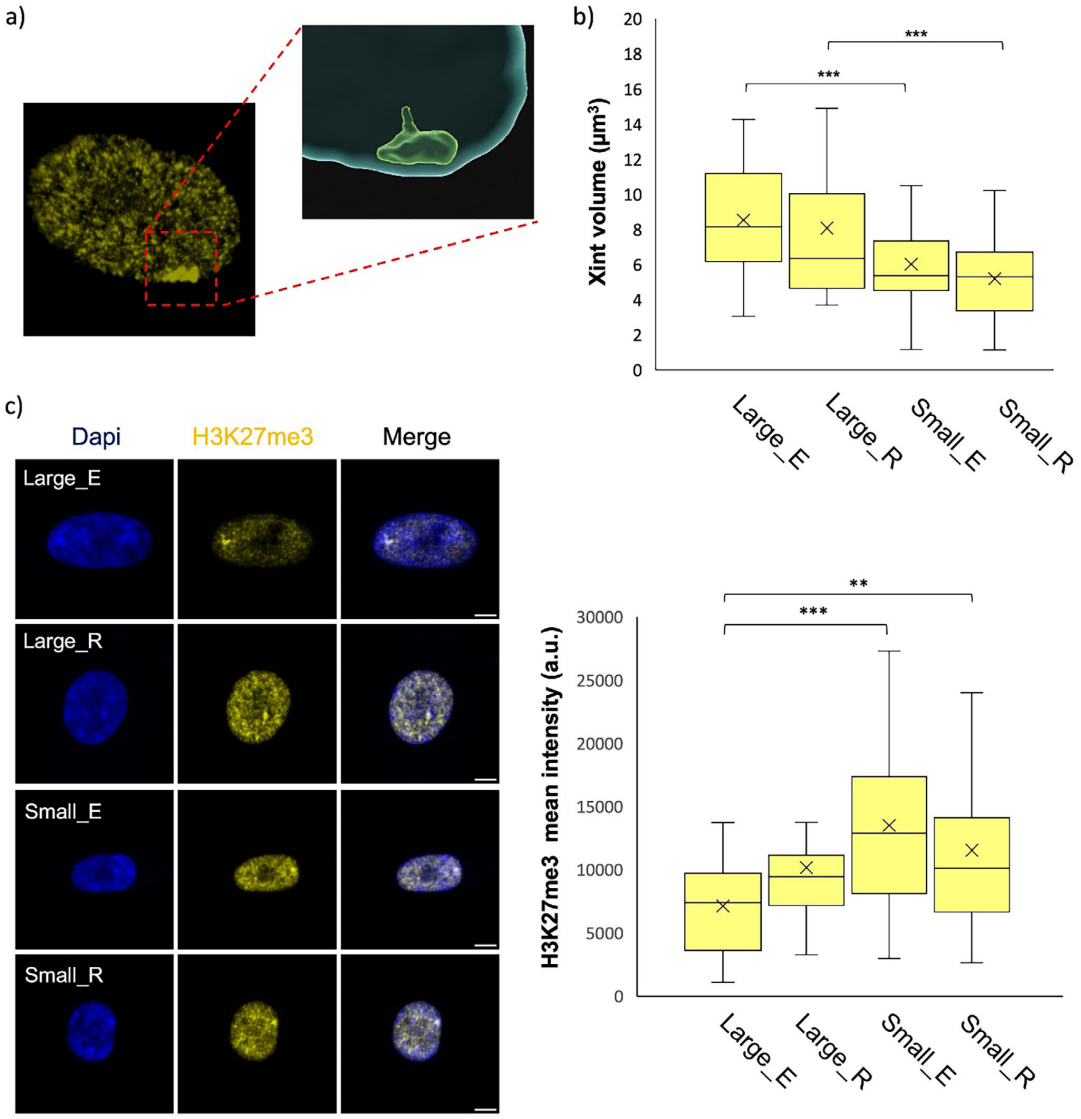
Induced‐nuclear deformations influence H3K27me3 condensation state. (a) Representative confocal images and 3D surface reconstruction of Chromosome X inactivated (Xint). (b) Box Plots of Xint volume for ASCs cultured on micropatterned surfaces. (c) Representative confocal images of immunofluorescence staining of nuclei (blue) and H3K27me3 (yellow) for all tested conditions. The scale Bar is 5 µm. Quantification of H3K27me3 intensity levels per nuclear volume for each tested condition. n = 40 nuclei for each condition from three independent experiments were calculated. Kruskal‐Wallis, Post‐Hoc Dunn's test (^*^ *p*< 0.05; ^**^ *p*< 0.001; ^***^ *p*< 0.0001).

To evaluate the effect of nuclear deformation on the condensation state of facultative heterochromatin, we performed confocal stacking on immunofluorescence‐stained cells for H3K27me3. Initially, we targeted the condensed heterochromatic regions known as the inactivated Chromosome X (Xint). Through confocal z‐stack reconstruction, we calculated the volume of the Xint (Figure 8a). We observed a progressive reduction in Xint volume from cells cultured on large adhesive islands to those cultured on small adhesive islands (Figure 8b), consistent with the trend of the nuclear volume data and in agreement with the CTs 12 and 6 volume evaluation (Figures 2 and 3). Xint volume was highest on Large_E and lowest on Small_R. Finally, we calculated the mean fluorescence intensity of H3K27me3 within the nuclear region for all tested conditions.

We also found that Large_E ASCs displayed a lower H3K27me3 nuclear content with respect to Small ASCs (Figure 8c). However, no difference was observed between Large ASCs groups (ie, Large_E vs. Large_R). These results suggested that facultative heterochromatin, more than constitutive ones, was less sensitive to deformation experienced by oblate nuclei of Large ASC. In conclusion, the data collectively demonstrate that deformations experienced by ASC nuclei could induce a reorganization of nuclear heterochromatin content. Large_E ASCs, possessing oblate and elongated deformed nuclei, exhibited a global chromatin decondensation state compared to other experimental groups.

## 3 Discussion

In this work, we investigated how material‐induced changes in cell and nuclear shape drive the reconfiguration of genetic matter, specifically focusing on chromatincondensation/decondensation state and the positioning of selected CTs. Changes in chromatin structure have been associated with the onset or progression of several biological processes, including differentiation [2], reprogramming [20], and tumorigenesis [38]. Indeed, there has been an increasing appreciation from the scientific community about the interplay between nuclear mechanical constraints and chromatin organization, with recent studies shedding light on how mechanical cues can influence the compaction and accessibility of chromatin fibers [7, 20, 21]. For instance, millisecond deformation experienced by the cell nucleus through microfluidic channels constriction resulted in a stable and sustained modification of heterochromatin organization [20]. High amplitude mechanical stretch deforming nuclei of endothelial progenitor cells activated a transient fluidification of heterochromatin that reduced the direct propagation of forces to the DNA. In contrast, low amplitude stretching led to a stable decondensation of nuclear heterochromatin content [21]. Some aspects of the nucleus and chromatin states have been shown over the past decade to correlate with cell spreading, but the mechanism through which cell mechanics influence chromatin condensation state remains unclear. The actin cytoskeleton plays an active role in the transmission of mechanical signals, with its assembly and mechanics regulating nuclear morphology and shape [9, 23, 26, 39]. Here, we utilized micro‐patterned surfaces capable of modulating cell stiffness and nuclear shape deformation through cytoskeleton‐generated force. Specifically, single‐cell micropatterning allowed us to coherently and homogeneously set the cell cytoskeleton in a quasi‐steady‐state conformation, thereby inducing defined and temporally stable mechanical deformation of the nuclear membrane. This enabled us to force the nuclear envelope to acquire specific shapes, ranging from prolate to oblate (Figure 2) and to investigate how chromatin adapts to prolonged mechanical constraints in terms of condensation and spatial organization. These different nuclear contours are accompanied by a different mechanical stiffness of the apical perinuclear region which, in turn, correlated with different organization and assembly of FAs plaques and actin stress fibers (Figure 1; Figures S2 and S3). ASCs cultured on large adhesive islands (Large_E and Large_R) exhibited a stiffer apical nuclear region compared to cells cultured on smaller adhesive patterned surfaces. This observation aligns with literature data, which indicates that an increase in cell size corresponds to the formation of a more structured network of actin filaments, resulting in increased cell stiffness [29, 31]. The elongated adhesive geometry further accentuated cell stiffening compared with the round counterpart, as shown in Figure 1a. The stiffening of the apical nuclear region can be ascribed, at least partly, to a thickening of actin cap fibers. Furthermore, elongated ASCs exhibited distinct organization of actin cap fibers above the cell nucleus (see Figure 1c). Actin‐cap fibers are known to regulate cell nucleus shape and mechanotransduction processes [40, 41], and ASCs cultured on different adhesive islands exhibited various distributions of these structures. Although we observed a relationship between perinuclear Young's modulus and mean F‐actin intensity when comparing Large and Small elongated ASCs, no significant difference in mean F‐actin content above the nuclear region was detected between stiffer Large_R and softer Small_R ASCs. The difference in the higher Young's modulus measured in ASCs cultured on large and small circular micropatterns may be attributed to the presence of a well‐organized actin cap. (see Figure 1e; Figure S3). In fact, in accordance with previous findings, cells with a developed actin cap are stiffer than cells without a cap [42]. The differences in cytoskeleton force intensity and their spatial distribution are likely to induce distinct morpho‐physical features of the cell nucleus (Figure 2). It has been demonstrated that cell elongation correlates with increased tension in central actomyosin cables, which regulate nuclear shape by transferring lateral compressive forces to the sides of the nucleus' [39]. Our data show that cellular elongation affects nuclear morphologies in relation to micropatterning size and ASCs spreading area. (Figure 2). Furthermore, a reduction in perinuclear stiffness induced a gradual increase in nucleus height (Figure 2d). The actin cap was found to mediate force transfer from the cytoskeleton, leading to a more effective vertical squeezing of the nucleus. Indeed, pharmacological treatments with drugs inhibiting actin cytoskeleton contractility led to an increase in nucleus height, indicating a less effective force transfer [41]. Accordingly, in small circular ASCs, the formation of a weaker actin cap led to a decrease in tension acting on the nuclear membrane, promoting the formation of thicker nuclei with respect to other experimental groups. Finally, our data show that deformations experienced by ASCs nuclei are accompanied by dynamic unfolding of the nuclear lamina (Figure 2h). During the nuclear flattening, lamins proteins undergo reorganization around the nuclear periphery, transitioning from an unfolded state in flattened nuclei to a wrinkled state in round nuclei [43].

The distinct nuclear shapes imposed by culturing ASCs on different substrates induced a corresponding rearrangement of nuclear genetic material. We observed that nucleus deformation influenced the organization of two chromosomal territories, CT6 and CT12, with their volumes scaling according to changes in nuclear volume. Chromosomes 6 and 12 represent an interesting pair for comparative analysis, as they differ substantially in size and gene density, two key determinants of nuclear localization [44, 45]. According to literature reports, our data indicate that cell geometry and cytoskeleton reorganization influence the repositioning and reorientation of chromosome territories along with their morphological features [34]. However, we noticed that nucleus deformation can impact different CTs positioning differently, with CT6 being more responsive to nuclear deformation than CT12. Similarly, Tsimbouri et al. observed statistically significant differences in the inter‐territory distance of CT1 in mesenchymal stem cells cultured on topographic nanopatterned surfaces compared to those cultured on flat control surfaces [46]. Furthermore, Lui et al. observed a translocation in the positioning of chromosome territories 18 and 19, along with alterations in the gene expression profiles of HeLa cells, following nuclear deformation of cells cultured on micropillar‐patterned surfaces [47]. In our setup, the position of CT12 pairs did not change significantly upon nuclear deformation, suggesting that not all CTs could be repositioned upon nuclear mechanical strains, as the deformation position is dependent. The capacity to modulate chromosome territories (CTs) positioning within the nucleus introduces a compelling scenario in which such re‐localization might promote the movement of CTs from densely packed peripheral regions toward more decondensed heterochromatin domains, generally located near the nuclear interior. This spatial reorganization could, in turn, influence local chromatin accessibility and potentially impact gene regulation. Besides the CTs organization, our data demonstrated that cytoskeleton‐induced nuclear deformation had a profound impact on chromatin condensation state. The role of cell size and nuclear volume on chromatin compaction states was already reported in the literature. More specifically, a decrease in cell spreading area and nuclear volume generally correlates with a condensation of chromatin content within the cell nucleus [31, 39, 48]. Consistent with literature reports, our experimental data suggested that the prolate nuclear morphology with smaller nuclear volumes was characterized by higher levels of both constitutive and facultative heterochromatin. Furthermore, with the 3D FE models, the chromatin organization can be correlated with nuclear volume changes (Figure S7a) and strain fields developed on the nuclear surface (Figure 3d). In particular, on Large micropatterned substrates, ASC nucleus on both elongated and round configurations have high von Mises equivalent strain with respect to the Small micropatterned counterpart, and also, the strain distributions are in agreement with volume profiles. This aligns with the model proposed by Farifasei et al., indicating that nucleo‐cytoplasmic shuttling of transcription and epigenetic factors occurred in an actomyosin‐dependent translocation [49]. Here, the reduced volumetric expansion we observed in Small ASCs is likely to reduce the influx of the enzyme histone deacetylases (HDAC) that leads to a global chromatin condensation. Therefore, actomyosin‐dependent translocation of these factors induced for stable modification of nuclear chromatin content.

**FIGURE 9:**
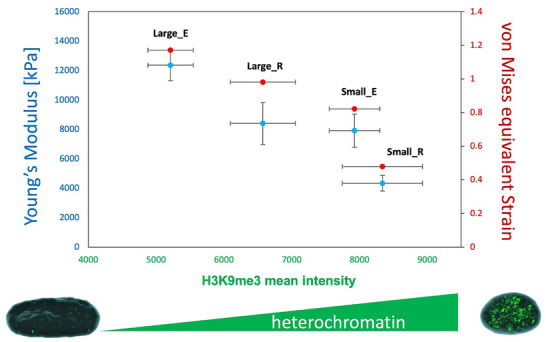
Increased cell stiffness and nuclear strain promote heterochromatin unfolding, facilitating the transition towards a more relaxed state.

Even though we did not monitor nuclear volume changes dynamically, heterochromatin decondensation is accompanied by local volumetric expansion. Our FEM simulation predicts that nuclei of Large ASC undergo more pronounced volumetric expansion and deformation during cell spreading (Figures S7a and S8) and hence are more prone to promote heterochromatin—euchromatin transition. Also, larger expansion is generally found at the cell periphery, events that are in principle compatible with the decreased intensity of H3K9me3 observed in those locations (Figure 7e). These local volume changes are a key outcome of the model, as they identify where, within the nucleus, mechanical deformation is most permissive to local compaction or dilation, providing a direct link between externally imposed cell shape constraints and spatially heterogeneous nuclear mechanics. This spatial resolution cannot be determined experimentally and represents a critical step toward interpreting the observed region‐specific chromatin decondensation. Accordingly, our experimental measurements consistently report nuclei of Large ASCs being poorer in both facultative and constitutive heterochromatin, and that H3K9me3 foci are less bright and more scattered with respect to nuclei of Small ASCs. Altogether, these observations suggest that extensive nuclear strains ultimately promote heterochromatin foci unfolding and facilitate the transition to a more relaxed state (Figure 9). However, we also found that Large_E ASCs exhibited lower levels of H3K9me3 compared to their Large_R ASCs counterparts, where no statistical difference was observed in terms of nuclear volume. These findings suggest that under conditions of increased nuclear volume, the condensation state of heterochromatin may also be regulated by the deformation of the cell nucleus caused by actin stress fiber assemblies. It was shown that the stem cells' nucleus in transition to differentiation expands when cells are stretched, suggesting an auxetic behavior [50]. Here, the auxetic phenotype of ESC nuclei is driven at least in part by global chromatin decondensation. In addition, modeling the nucleus as a compressible chromatin polymer gel, it has been shown that the mechanical properties can be auxetic in the case of highly decondensed chromatin [51].

In addition, our data provides novel insight into how constitutive heterochromatin condensation occurred upon nucleus deformation. More precisely, a variation of the number and the size of heterochromatin foci, along with their distribution, occurs within different nuclear deformation cases. Specifically, more oblate nuclear shapes exhibit a higher number of heterochromatin foci, yet simultaneously display a lower degree of chromatin condensation compared to those with a more prolate shape. Here, a reduction of stresses acting on nuclear membrane resulted in: (i) a reduction of the number of heterochromatin foci; (ii) an increase of H3K9me3 foci size (iii) an increase of the interdistance between heterochromatin foci; (iv) an increase of the percentage of foci located toward nuclear periphery; (v) an increase of their fluorescence intensity; (vi) a reduction of the distance from nuclear lamina. An aggregation of such foci during chromatin condensation could be an explanation of our results. This observation is in agreement with previous findings where changes in the number and size of H3k9me3 clusters occur during chromatin condensation, resulting in a reduction of their number and the formation of larger, more compacted nanoclusters [52]. Nucleus deformation also affects H3K9me3–nuclear lamina interaction, where stretch‐induced chromatin decondensation reduced H3K9me3‐marked lamina‐associated heterochromatin [21]. Taken together, our findings support the notion that heterochromatin foci fragmentation and aggregation can be finely tuned by variations in nuclear tension. This points to a mechanically driven regulation of heterochromatin organization, whereby forces acting on the nuclear membrane contribute to shaping both the architecture and condensation state of H3K9me3‐enriched chromatin domains. This is crucial since H3K9me3 proved to inhibit RNA polymerase II recruitment to the promoter site, thus blocking force‐induced transcription activation, particularly near the nuclear periphery. However, its pharmacological down‐regulation increases force‐induced transcription of a mechano‐nonresponsive gene, FKBP5 (FKBP prolyl isomerase 5) located at the nuclear periphery [53]. Thus, the understanding of the mechanism regulating heterochromatin condensation state is crucial to control mechanical transcription upregulation of endogenous genes otherwise not expressed. A stable and selective activation of such genes could, in principle, reprogram cellular transcriptional activity, ultimately leading to a modification of cellular function and fate.

## 4 Conclusion

In summary, our results shed light on how material‐induced nuclear deformation affects heterochromatin organization and distribution. We demonstrated that tuning cell mechanics enables the modulation of nuclear morphology and heterochromatin organization. Specific assemblies of the cytoskeleton induce cells to acquire very different nuclear shapes ranging from oblate to prolate ellipsoids. Additionally, the modulation of nuclear shape profoundly affects chromatin conformation, with prolate nuclei being richer in heterochromatin with respect to oblate ones. Notably, a shift toward a more oblate and elongated nuclear morphology correlated with chromatin decondensation in ASCs. Our results suggest a novel approach for mechanically instructing cells to control and guide their fate. The engineering of mechano‐modulating culturing platforms could be pivotal in developing a novel approach aimed at controlling gene expression patterns through mechanical conditioning of the stem cell nucleus. Understanding the mechanisms underlying the mechanical activation of genetic sequences could lay the groundwork for a novel paradigm of cellular programming.

## 5 Experimental Section/Methods

### 5.1 Substrate Fabrication and Functionalization

Fabrication of culture substrates possessing microscopic features in the form of single‐cell adhesive islands was made using the UV‐deep light micropatterning technique according to the protocol developed by Azioune et al. [54]. A single‐cell micropatterning culturing platform was designed with adhesive islands separated by 50 µm from each other, to avoid cell‐cell contact, thus avoiding the jump of a cell from an adhesive island to the closest one. Specific geometric characteristics of the adhesive islands were reported in Figure S1. Briefly, glass coverslips were washed in 70 % ethanol for 10 min and dried. Then, they were first activated by exposure to O2 plasma (PFEIFFER VACUUM SingleGauge—CESAR RF Power Generator) for 1 min and incubated for 1 h with 0.1 mg mL−1 poly‐l‐lysine‐g‐poly(ethyleneglycol) (PLL‐(20)‐g[3.5]‐PEG(2); SuSos Company) in 10 mM HEPES at pH 7.3 for passivation. After washing with distilled water, the treated surfaces were illuminated with deep UV light (ProCleaner Plus, BioForce Nanosciences) through a chromium synthetic quartz photomask (4DCell), possessing the desired micropattern feature (Figure S1), for 5 min to selectively burn desired PLL‐g‐PEG regions.

### 5.2 Cell Culture

Immortalized adipose‐derived Mesenchymal Stem Cells (ASC52telo, hTERT; ATCC) in passages four to eight were cultured in Mesenchymal Stem Cell Basal Medium (ATCC) supplemented with mesenchymal stem cell growth kit for adipose and umbilical‐derived MSCs—Low Serum (ATCC) at 37°C in a humidified atmosphere of 95 % air and 5 % CO2; the culture medium was changed every 2 days. After 3 days of culture, cells were detached with trypsin (Gibco, Thermo Fisher Scientific, Waltham, MA, USA) and seeded on all patterned substrates at a density of 4000 cells/cm^2^. Prior to cell seeding, all patterned and reference substrates were incubated for 1 h with 50 µg/mL fibronectin solution (Sigma–Aldrich, St. Louis, MO, USA) at room temperature, followed by two washes in PBS1x.

### 5.3 Cell Fixation and Immunostaining

Cells cultured on all patterned substrates were fixed 24 h after seeding with 4 % paraformaldehyde (Alfa Aesar‐Thermo Fisher, Karlsruhe, Germany) in PBS for 15 min. The cells were then permeabilized with 0.1 % Triton X‐100 (Sigma–Aldrich‐Merck KGaA, Darmstadt, Germany) in 1x PBS (TPBS). Samples were blocked in 3 % bovine serum albumin in PBS (Sigma–Aldrich) for 1 h to avoid non‐specific binding. Samples were subsequently incubated overnight at 4°C with primary antibody in 3 % BSA/0.1 % Triton X‐100/PBS, followed by washing in PBS and incubation with secondary antibody in 3 % BSA/0.1 % Triton X‐100/PBS for 1 h. Constitutive and facultative heterochromatin were labeled by incubating samples with an anti‐H3K9me3 monoclonal antibody (dilution 1:200, Cell Signaling Technology) and an anti‐H3K27me3 monoclonal antibody (dilution 1:200, Cell Signaling Technology), respectively. Nuclear Lamina was labeled by incubating samples with anti‐Lamins A/C monoclonal antibody (dilution 1:500, SantaCruz Biotechnology). Actin filaments were stained by incubating samples with Phalloidin (dilution 1:250: Cytoskeleton Inc) for 1 h at 20°C. Nuclei were stained by incubating samples with DAPI (4’,6‐Diamidino‐2‐Phenylindole, Dihydrochloride, Thermo Fisher Scientific) solution (dilution 1:10 000) for 15 min. Samples were thoroughly rinsed in PBS and mounted on glass slides by using Vectashield Antifade Mounting Medium (Vector Laboratories, Cat. H‐1000‐10). Fluorescent images of nuclei, actin bundles, and chromatin were collected with a Zeiss Confocal LSM 700 microscope using a 63X objective. Samples were excited at 380 nm (Nuclei), 488 nm (H3K9me3/H3K27me3), and 543 nm (Actin), and the emissions were collected in the 400–420 nm, 500–530 nm, and 560–590 nm ranges, respectively. Z‐stacks were acquired with the optimal interval suggested by the software.

### 5.4 Chromosome Painting

Fluorescent in situ hybridization was performed using a protocol enabling 3D nuclear structure preservation. Briefly, cells were fixed with 4 % PFA for 10 min. Then, the specimens were incubated for 24 h in 20 % glycerol/1× PBS, followed by freeze‐thawing cycles in liquid nitrogen. The cells were permeabilized in 0.07 % Triton‐X/1xPBS/0.1 M HCl for 10 min, and DNA was denatured in 50 % Formamide/2xSSC (pH = 7.4) for 10 min. Then, chromosome painting probes (Metasystems, Xcyting Chromosome Paints) were added to the specimen, denatured for 3 min at 75°C, and hybridized at least 16 h at 37°C in hybridization chamber. Afterward, the cells were washed for 10 min in 2xSSC and 0.1SSC buffers. Nuclei were stained with DAPI (Sigma–Aldrich Cat. D8417), and the samples were mounted in Vectashield Antifade Mounting Medium (Vector Laboratories, Cat. H‐1000‐10).

### 5.5 Fluorescence Lifetime Imaging

Measurements of the fluorescence lifetime of Hoechst 33342 on fixed cells were performed on a Leica TCS SP5 II SMD inverted laser scanning confocal microscope equipped with a Ti:sapphire mode‐locked, 80 MHz, 150 fs pulse‐width, pulsed near infrared Coherent Chameleon Ultra 2 laser for 2‐photon (2P) excitation. Fixed samples were first incubated with glycine 20mM for 10 min and then with Hoechst 33342 (dilution 1:10 000) for 15 min at room temperature. Time domain FLIM was performed by using a Becker & Hickl Time Correlated Single Photon Counting (TCSPC) FLIM system (SPC‐182NX, 2‐channel module with HPM‐100‐07 hybrids detector) connected to the NDD (Non Descanned Detector) port of the Leica confocal microscope. Hoechst 33342 was 2P‐excited at 820 nm, and the emission was detected in the 400–470 nm wavelength band. Fluorescence lifetime data were acquired and analyzed by Becker & Hickl's software, respectively, SPCM and SPCImage NG. The maximum‐likelihood (MLE) algorithm was used for fitting TCSPC experimental data with decay functions (sums of exponential functions of different decay times) to calculate lifetimes and amplitudes. The goodness of fitting was verified by the chi2 value and analysis of the residual graph. Segmentation of nuclear regions to be analyzed was performed by using the phasor plots tool available in the SPCImage software. In brief, decay data in the individual pixels are expressed as phase and amplitude values in a polar diagram [55]. Independent of their location in the image, pixels with similar decay signatures form clusters in the phasor plot. Different phasor clusters can be selected, and the corresponding pixels back‐annotated in the FLIM images. Clusters representing nuclear regions were selected, and the decay functions of the pixels within the selected phasor range were combined into a single decay curve of high photon number. These curves were analyzed at high accuracy, revealing the lifetime decay components [56]. Heat maps were generated to observe the lifetime distribution. For treatment‐to‐treatment comparison, we quantified changes by calculating the mean fluorescence lifetime in the whole nuclei.

### 5.6 Atomic Force Microscopy

JPK NanoWizard II AFM was used to measure the mechanical properties of living cells. Measurements of cell mechanical properties were carried out in cell culture medium supplemented with 12.5 mM HEPES buffer (EuroClone) at 37°C. An optical microscope was combined with the AFM to visualize both the tip and the sample. Soft cantilevers (SAA‐SPH‐5UM, nominal spring constant 0.25 N/m) with a spherical tip of radius 5 µm were used to investigate cell mechanical properties. The AFM was operated in force spectroscopy mode. A total of 64 force indentations, covering 2 × 2 µm^2^ areas on the apical perinuclear region, were performed to measure cell stiffness (Young's modulus). Cell indentation depth was chosen to be equal to 50 nm. Evaluation of the Young's modulus was performed with JPKSPM data processing software. Specifically, the force indentation curves from each measurement were fitted with a Hertzian model to obtain the Young's modulus [24, 57].

Results were reported as boxplots showing the median, the first and third interquartile, and the minimum and the maximum values.

### 5.7 Image Analysis

Detailed methods for image analysis are provided in the Supporting Information.

### 5.8 Finite Element Simulations

Finite element (FE) simulations were performed using ABAQUS CAE 2025 (Dassault Systémes Simulia Corp.). In the 3D FE model, the cell was assumed to be a structure composed of nucleus, cytoplasm, and actin cap. Both cell and nucleus were modeled as compressible isotropic Neo‐Hookean hyperelastic materials, each with distinct mechanical properties. The compressibility of these materials was accounted for due to observed volume changes under large displacement fields [58, 59]. The Young's modulus, E, was set to 4000 Pa for the nucleus and 750 Pa for the cytoplasm [58, 60, 61, 62], while Poisson's ratio, ʋ, was assigned a value of 0.25 and 0.35 for the nucleus and cytoplasm, respectively [58, 63]. A perfect connection at the nucleus‐cytoplasm interface was assumed [58, 64]. Finally, the glass micropatterned substrate was modeled as a linear incompressible elastic material with E of 50 GPa.

In the initial configuration, the cytoplasm and nucleus were assumed as two perfectly concentric 3D spheres with a diameter of 20 µm and 9.7 µm, respectively. These dimensions were derived from experimental images of cells in an undeformed configuration. Detailed, the undeformed configuration corresponds to cells seeded on a micropatterned glass substrate and fixed after 5 min. Particularly, this time marks the early stage of the spreading process with cells that still have a spherical shape. The external part of the cell was perfectly in contact with the glass substrate in order to prevent the cellular penetration in the pattern during the spreading process.

Cytoplasm, nucleus, and substrate were all discretized using the same element type: a first‐order solid continuum element with 8 nodes and a hybrid formulation (C3D8H). The element size for the cytoplasm and nucleus was 670 nm, resulting in a total of 31322 solid elements and 34636 nodes for the entire model. For each experimental scenario, a 3D finite element simulation was performed to reproduce the stretching of the whole cell on the substrate. This was achieved by applying an experiment‐derived displacement field in cylindrical coordinates to the cytoplasmic surface (see Supporting Information—The 3D Finite Element Model Approach). The influence of the actin cap on the nuclear surface was simulated by implementing distributed nodal forces over a specific region of interest, corresponding to the observed extent. This force was quantified, knowing the force (3 nN) exerted by a single actin cap stress fiber on the nucleus [65], the number of stress fibers, and the affected surface of interest, both determined experimentally. Consequently, the distributed force values corresponded to 15 nN for Small_E, 27 nN for Large_R, and 30 nN for Large_E micropatterns. Furthermore, since no actin cap formation was observed in the Small_R configuration, no force was applied.

### 5.9 Statistical Analysis

Significant differences in the data obtained from image analysis were assessed using the Kruskal–Wallis test followed by Dunn's multiple comparisons post‐hoc test (^*^ *p*< 0.05; ^**^ *p*< 0.001; ^***^ *p*< 0.0001), performed in Origin. Statistical analyses were also performed using linear mixed‐effects models to account for repeated measures arising from the mechanical mapping. The fixed effect is represented by the shape of the adhesive area, and random effects were specified for individual cells. Models were fitted using MATLAB. Each experiment was repeated three times, and at least n = 15 cells/nuclei for each condition were acquired.

## Author Contributions

Carlo F. Natale and Paolo A. Netti designed the study. Carlo F. Natale performed the experimental work and analyses. Luca Messina contributed to the characterization of chromosome territories and spatial distribution analysis. Valeria Panzetta contributed to the characterization of cell mechanical properties. Stefania Saporito and Costantino Menna developed 3D finite element models. Maurizio Ventre contributed to conceptualization, writing, review, and editing. Carlo F. Natale, Luca Messina, Valeria Panzetta, Stefania Saporito, Costantino Menna, Maurizio Ventre, and Paolo A. Netti wrote and edited the manuscript.

## Conflicts of Interest

The authors declare no conflicts of interest.

## Supporting information




**Supporting File**: advs74158‐sup‐0001‐SuppMat.docx.

## Data Availability

Data will be made available on request.
