# Supplementary material for: Material‐Induced Nuclear Deformation Controls Chromatin Architecture in Adipose Stem Cells
Source: Adv Sci (Weinh). 2026 Jan 30;13(30):e14458. doi: 10.1002/advs.202514458 (PMC13248850; doi:10.1002/advs.202514458)
Supplement: Supplementary file 1 — Supporting File: advs74158‐sup‐0001‐SuppMat.docx. [file ADVS-13-e14458-s001.docx]

Supporting Information

Material-Induced Nuclear Deformation Controls Chromatin Architecture in Adipose Stem Cells

Carlo F. Natale, Luca Messina, Valeria Panzetta, Stefania Saporito, Costantino Menna, Maurizio Ventre, Paolo A. Netti*

**Materials and Methods**

**Image Analysis**

Cellular spreading was evaluated with the command “Measure” of Fiji. At least 20 confocal digital images were randomly collected and analyzed for each tested condition from two independent experiments. Actin-nucleus orientation was determined using custom mad script in ImageJ where the orientation of the actin cytoskeleton was determined with OrientationJ plug-in and nuclear major axis orientation was determined with analyze particles command.

Morphometric analysis (area, length and orientation) of FAs was performed in Fiji. Confocal digital images of FAs were first processed using the ‘blur’ command by following a modified version of the procedure proposed by Maruoka et al. ^[1]^ Blurred images were subtracted from the original images using the image calculator command. The images were further processed with the ‘threshold’ command to obtain binary images. Then particle analysis was performed to extract the morphometric descriptors. Only FAs whose area was above 1 µm^2^ were included in the statistical analysis.

To extract and generate distribution maps of actin caps, confocal z-stacks of nuclei (DAPI) and actin cytoskeleton (phalloidin) were acquired for single cells. A schematic representation of this image analysis methodology is provided in Figure S13*.* More specifically*,* each z-stack was first translated and rotated to align the nuclear centroid and the principal nuclear axes across all cells, ensuring consistent orientation. To specifically analyze only the actin cap and exclude basal actin fibers, the z-stack was divided into basal, central, and apical sections, using the nucleus as a reference. This division was based on identifying the basal (s_nucleus) and apical (S_nucleus) nuclear slices (i.e. where the nuclear fluorescence was barely visible). The total number of optical slices spanning the nucleus was defined as Δ, and this range was subdivided into three equal segments of thickness δ = Δ/3. The basal region corresponded to all sections s below the lower third of the nucleus (s <= s_nucleus + δ). The remaining two-thirds, comprising the central and apical parts of the cell, were combined and defined as the supranuclear region (s > s_nucleus + δ), ensuring that the entire actin cap above the nucleus was captured in the analysis. Only this region was used for subsequent analysis. The actin-cap area within this region was defined by fitting an elliptical mask to the 2D nuclear projection and then enlarging the mask‘s major and minor semi-axes by 25 % including both the nuclear and perinuclear areas. This enlargement provided a margin that minimized potential edge effects visible in the final maps and ensured that the analyzed area encompassed both the nuclear and immediately surrounding perinuclear zones where the actin cap is organized. Using this elliptical ROI, only the portion of the z-stack corresponding to the actin cap was extracted. Each actin-cap substack was then projected along the z-axis to obtain a single 2D image representing the actin distribution for that cell. The individual actin-cap projections from all analyzed cells were subsequently aligned to the nuclear centroid, overlaid, and summed to generate averaged, condition-specific distribution maps (*n* = 10 cells per condition). Fluorescence intensity values were normalized within each map between 0 and 1 to allow direct comparison across conditions All image processing was performed using a custom ImageJ macro derived from previously published approaches ^[2]^

Nuclear lamina texture was evaluated following the procedure proposed by Katiyar A, et al. ^[3]^ Briefly, Lamins A/C confocal images were firstly segmented with the Otsu algorithm and filtered to remove small artifacts. Features in the nuclear lamina were then detected using a Laplacian of Gaussian filter. The algorithm excluded contributions from the nuclear envelope, first by identifying its boundary using the Sobel edge detection algorithm and then subtracting it from the texture-detected image. Finally, the texture pixels within each nucleus were counted to yield a total number.

Nucleus morphological parameters were extracted from confocal z-scanning using Imaris 12.1 software (Andor Technology). A nuclear surface was created from the binary z-stack images using surface command of Imaris software. More specifically, a surface surrounding the entire nucleus was created based on DAPI fluorescence intensity and then this object was used to extract information of morphological feature of the surface (ellipticity, volume, thickness, length and width) along with fluorescence intensity for every fluorescence channel acquired for the experiment.

To identify nuclei in the G1 phase, we followed a procedure proposed by Pere Rocha et al. ^[4]^ Briefly, we plotted the histogram of the DNA content of the entire cell population calculated as the total fluorescence intensity of emitted by each nucleus. Then, the histogram showed two peaks corresponding to cells in G1 and G2. To select cells in G1, nuclei with DNA content higher than the center of the first peak plus 1.5 standard deviations were discarded.

To describe the chromosomal territories organization in the 2D nuclear space, we adopted the approach developed by Iver et al ^[5]^ based on Euclidean geometry computing the chromsomomal homologous interdistance (ID) and the Interphysical Distance (IPD). The ID refer to the Euclidean distances between the centroids of homologous chromosomes within the same nucleus (i.e., CT12A with CT12B, and CT6A with CT6B). These distances were calculated using the following 2D Euclidean distance formula:

$$ID_{CT12} = \sqrt{\left( x_{12A} - x_{12B} \right)^{2} + \left( y_{12A} - y_{12B} \right)^{2}}$$

$$ID_{CT6}=\sqrt{\left( x_{6A}-x_{6B} \right)^{2}+\left( y_{6A}-y_{6B} \right)^{2}}$$

Here, $\left( x_{12A} , y_{12A} \right)$ and ($\left( x_{12B} , y_{12B} \right)$ represent the coordinates of the centroids for homologous chromosome 12, while $\left( x_{6A} , y_{6A} \right)$ and $\left( x_{6B} , y_{6B} \right)$ are the centroids for chromosome 6. The notation A and B is arbitrary and serves to differentiate the homologous chromosomes for the purposes of distance calculations. The IPD is defined as the mean Euclidean distance between all possible pairwise combinations of centroids from chromosomes 6 and 12 (i.e., CT12A with CT6A, CT12A with CT6B, CT12B with CT6A, and CT12B with CT6B). The IPD was calculated using the following formula:

$$IPD=\frac{1}{4} \left( \sqrt{\left( x_{12A} - x_{6A} \right)^{2} + \left( y_{12A} - y_{6A} \right)^{2}} + \sqrt{\left( x_{12A} - x_{6B} \right)^{2} + \left( y_{12A} - y_{6B} \right)^{2}} + \sqrt{\left( x_{12B} - x_{6A} \right)^{2} + \left( y_{12B} - y_{6A} \right)^{2}} + \sqrt{\left( x_{12B} - x_{6B} \right)^{2} + \left( y_{12B} - y_{6B} \right)^{2}} \right)$$

For heterochromatin mean intensity quantification, values were normalized with respective nuclear volume. A surface around the heterochromatin (H3K9me3) was created with Surface commands and the ratio with respect to total nuclear volume was calculated. Imaris "Spots" function was then utilized to tag each heterochromatin aggregate (foci) enclosed in the nuclear surface with spots of 0.8 μm diameter, providing insights into the spatial distribution of this epigenetic marker and quantifying the number of H3K9me3 fluorescent aggregates per cell. To estimate H3K9me3 foci size we employed the same approach used to track H3K9me3 foci centroids, whereby spheres of variable diameter were generated around each heterochromatin focus, with sphere size adapting to the local fluorescence contrast by means of Imaris Spots (Region Growing) function*.* Heterochromatin domains interdistances were calculated with 3 neighbours Average distances command and their distance from nuclear lamina was calculated with smallest surface distances commands. For CTs analysis, a surface surrounding the entire CT was created based on Chromosome Painting fluorescence intensity with Imaris Surface and Spot function. The coordinates of two pairs CTs centroids for each experimental condition were extracted with Spot functions in Imaris.

The spatial distribution of heterochromatin spots within cellular nuclei was analyzed by quantifying the radial distance of the spots from the nuclear centroid, defined as the geometric center of the nucleus In particular, the heterochromatin distribution in the nuclear space has been evaluated by means of an adapted version of the erosion analysis, which include dividing the nucleus into six concentric three-dimensional shells, and classifying each heterochromatin foci in accordance with the shell in which they are located, allowing a methodical and repeatable evaluation of the spots' positions relative to the nuclear center. This method has been largely used to assess the spatial distribution of nuclear elements, such as heterochromatin ^[6]^ and chromosomes ^[7]^, within cell nuclei. However, before proceeding with the classification, it was necessary to standardize the orientation of each nucleus. We aligned the nuclei relative to a common reference system, ensuring that each nucleus was uniformly oriented with respect to the others. Subsequently, we geometrically transformed the shape of the nuclei from elliptical to spherical to facilitate classification based on the shells and comparative analysis between different samples. In detail, given the inherently elliptical shape and random orientation of the nuclei relative to the global reference system, designated as $O-xyz$, it was essential to standardize the orientation of each nucleus with respect to a common reference. For this purpose, we introduced a local reference system for each nucleus, designated as $O’-x’y’z’$ with the origin positioned at the nuclear centroid and the axes $\bar{X’}$, $\bar{Y’}$ and $\bar{Z’}$ aligned respectively with the minor semiaxis $\bar{A}$, major semiaxis $\bar{B}$, and orthogonal semiaxis $\bar{C}$ of the specific ellipsoidal nucleus. The alignment process involved two crucial phases: translation and rotation. The global reference system $O-xyz$ was translated to coincide the origin $O$ with that of the nuclear reference system $O’$. The coordinates of a generic point $p(x,y,z)$ in the global system were transformed into the translated one as follows:

$$p_{t}(x_{t},y_{t},z_{t})=p(x-x_{c}, y-y_{c}, z-z_{c})$$

where $C(x_{c},y_{c},z_{c})$ represent the coordinates of the nuclear centroid expressed in the global reference system. We then applied a rotation matrix $\boldsymbol{R}$ to align the axes of the translated reference system $O_{t}-x_{t}y_{t}z_{t}$ with those of the nuclear system $O’-x’y’z’$ using the direction cosines of the nuclear ellipsoid axis evaluated through image analysis software (Imaris):

$$R=\left[ \begin{matrix} A_{x} & A_{y} & A_{z} \\ B_{x} & B_{y} & B_{z} \\ C_{x} & C_{y} & C_{z} \end{matrix} \right]$$

The coordinates of the translated point $p_{t}$ in the new rotated system were calculated as:

$$p_{r}(x_{r},y_{r},z_{r})=\boldsymbol{R}{\cdot p}_{t}(x_{t},y_{t},z_{t})$$

After aligning the nuclei, we applied a geometric transformation to convert the elliptical configuration into a spherical one, standardizing the dimensions and shapes of each nucleus:

$$p_{sph} (x_{sph},y_{sph},z_{sph}) = p_{r}\left( \frac{x_{r}}{A},\frac{y_{r}}{B},\frac{z_{r}}{C} \right)$$

Where A, B and C are the nuclear elliptical semiaxis lengths. Transforming the nucleus into a unitary sphere facilitated a homogeneous comparison of the coordinates of the heterochromatin spots transformed into spherical coordinates. After, we divided the nuclear space into six concentric spherical shells, each characterized by a specific external radius. The radii of the shells are defined as $R_{1},R_{2},\ldots,R_{s}$, with $R_{1}<R_{2}<\ldots<R_{s}=1$ and $R_{0}=0$ representing the center of the nucleus. These shells were designed to have identical volumes, ensuring that each heterochromatin spot had the same statistical probability of being positioned in any shell, assuming an originally uniform distribution of spots within the nucleus. This design ensures an unbiased assessment of spot distribution across the nucleus. To precisely define the concentric shells, we calculated the outer radii $R_{i}$ of each shell. The calculation of the radii is based on the following sequence of equations.

The volume $V_{0}$ of a single shell is given by the formula:

$$V_{0}=\frac{4}{3}\pi\frac{1}{s}$$

where $s$ is the total number of shells, in our case $s=6$. The volume of the i-th shell $V_{i}$ is proportional to the base volume $V_{0}$ given by:

$$V_{i}=i*V_{0}=\frac{4}{3}\pi{*R}_{i}^{3}=i*\frac{4}{3}\pi\frac{1}{n}$$

The radius $R_{i}$ of each shell, which corresponds to the outer radius of the i-th shell, is calculated from the formula of the volume of the sphere:

$$R_{i}=\sqrt[3]{\frac{{3V}_{i}}{4\pi}}$$

Finally:

$$R_{i}=\sqrt[3]{\frac{i}{n}}$$

Once the shells were established, each spot of heterochromatin was classified based on the shell in which it was located, assigning a number from 1 (outermost) to 6 (innermost). To classify the heterochromatin spots within these shells, we introduced a mathematical function $F$ that assigns each spot an integer value from 1 to 6 corresponding to the shell in which it is located. The position of each spot $p_{sph}(x_{sph},y_{sph},z_{sph})$ is defined in the coordinate system of the unitary sphere, and the radial distance $d$ from the center is calculated as:

$$d=\sqrt{\left( x_{sph}^{2}+y_{sph}^{2}+z_{sph}^{2} \right)}$$

The classification function $F$ operates according to the following logic:

$$F≝\left\{ \begin{aligned} 1, R_{0}\leq d<R_{1} \\ 2, R_{1}\leq d<R_{2} \\ \begin{aligned} \vdots\\ s, R_{s-1}\leq d\leq R_{s} \end{aligned} \end{aligned} \right.$$

This methodology allows us to systematically quantify and catalogue the position of the spots within the nucleus, providing a solid basis for comparative statistical analyses between different experimental samples. It is important to clarify that, to address edge effects arising from the irregular shape of the considered nuclei, a sphere with a radius 5% smaller than the unitary one was used. Any spot falling outside this adjusted sphere was classified as belonging to the outermost shell.

**The 3D Finite Element Model Approach**

To replicate cellular stretching observed in experiments on micropatterned substrates (Large_E, Large_R Small_E, Small_R,), each 3D finite element model was performed starting from the corresponding experimental setup. Specifically, a displacement field derived directly from experimental measurements and expressed in cylindrical coordinates, was applied. This field, formulated to achieve the final experimental cell morphology, drove the deformation of the cell, with the simulated nuclear shape emerging as a result of the displacements applied to the cell’s outer surface.

| **Large_E** | $u_{2} \left( \rho, \theta,z \right)$ | $0.006*\left( 1- \frac{z}{{9.231*10}^{-3}} \right)$ |
| --- | --- | --- |
|  | $u_{1} \left( \rho, \theta,z \right)$ | ${1.3*[-9\times10}^{11}z^{6}-1\times{10}^{10}z^{5}+9\times{10}^{7}z^{4}+1\times{10}^{6}z^{3}-6967z^{2}+48.024z+1.1751]*[0.019cos2\theta+0.02]$ |
| **Large_R** | $u_{2} \left( \rho, \theta,z \right)$ | $0.0039*\left( 1- \frac{z}{{9.231*10}^{-3}} \right)$ |
|  | $u_{1} \left( \rho, \theta,z \right)$ | ${0.021*[-9\times10}^{11}z^{6}-1\times{10}^{11}z^{5}+9\times{10}^{7}z^{4}+1\times{10}^{6}z^{3}-6967z^{2}+ 48.024z+1.1751] *[1-(0.18*\left\vert sin\vartheta\right\vert)]$ |
| **Small_E** | $u_{2} \left( \rho, \theta,z \right)$ | $0.005*\left( 1- \frac{z}{{9.231*10}^{-3}} \right)$ |
|  | $u_{1} \left( \rho, \theta,z \right)$ | ${0.7*[-9\times10}^{11}z^{6}-1\times{10}^{10}z^{5}+9\times{10}^{7}z^{4}+1\times{10}^{6}z^{3}-6967z^{2}+ 48.024z+1.1751]*[0.024cos2\theta+0.02]$ |
| **Small_R** | $u_{2} \left( \rho, \theta,z \right)$ | $0.0048*\left( 1- \frac{z}{{9.231*10}^{-3}} \right)$ |
|  | $u_{1} \left( \rho, \theta,z \right)$ | ${0.007*[3\times10}^{11}z^{6}-3\times{10}^{9}z^{5}+967043z^{4}+1\times{10}^{6}z^{3}-973.56z^{2}+ 56.437z+1.0971] *[1-(0.18*\left\vert sin\vartheta\right\vert)]$ |

**Table S1:** Experiment-derived displacement fields applied on the cytoplasmic surface to simulate the stretching of the whole-cell on each micropattern. *u*_1_ ($\rho$, $\theta$, z) and *u*_2_ ($\rho$, $\theta$, z) are the components along the vertical and radial direction, respectively, of the vectorial displacement field ***u*** ($\rho$, $\theta$, z) applied in the cylindrical coordinates.

**SI References**

1. M. Maruoka, M. Sato, Y. Yuan, M. Ichiba, R. Fujii, T Ogawa, N. Ishida-Kitagawa, T. Takeya, N. Watanabe N. Abl-1-bridged tyrosine phosphorylation of VASP by abelson kinase impairs association of VASP to focal adhesions and regulates leukaemic cell adhesion. *Biochem. J*. 441, 889–899 (2012).
2. P. Nastały, D. Purushothaman, S. Marchesi, A. Poli, T. Lendenmann, G.R. Kidiyoor, G.V. Beznoussenko, S. Lavore, O.M. Romano, D. Poulikakos, M.C. Lagomarsino, A.A. Mironov, A. Ferrari, P. Maiuri. Role of the nuclear membrane protein Emerin in front-rear polarity of the nucleus. *Nat Commun*. 11(1):2122 (2020).
3. A. Katiyar, V.J. Tocco, Y. Li, V. Aggarwal, A.C. Tamashunas, R.B. Dickinson, T.P. Lele. Nuclear size changes caused by local motion of cell boundaries unfold the nuclear lamina and dilate chromatin and intranuclear bodies. *Soft Matter*.15(45):9310-9317 (2019).
4. P. Poca-Cusachs, J. Alcaraz, R. Sunyer, J. Samitier, R. Farré, D. Navajas. Micropatterning of single endothelial cell shape reveals a tight coupling between nuclear volume in G1 and proliferation. *Biophys J*. 94(12):4984-95 (2008).
5. K.V. Iyer, S. Maharana, S. Gupta, A. Libchaber, T. Tlusty, G.V. Shivashankar. Modeling and experimental methods to probe the link between global transcription and spatial organization of chromosomes. PLoS One. 7(10):e46628 (2012).
6. S. Eck, S. Wörz, K. Müller-Ott, M. Hahn, A. Biesdorf, G. Schotta, K. Rippe, K. Rohr. Combined Model-Based and Region-Adaptive 3D Segmentation and 3D Co-Localization Analysis of Heterochromatin Foci. *Informatik aktuell*. 9-14 (2012).
7. N. Zuleger, S. Boyle, D.A. Kelly, J.I. de las Heras, V. Lazou, N. Korfali, D.G. Batrakou, K.N. Randles, G.E. Morris, D.J. Harrison, W.A. Bickmore, E.C. Schirmer. Specific nuclear envelope transmembrane proteins can promote the location of chromosomes to and from the nuclear periphery. Genome Biol. 14(2):R14 (2013).

**Figures**


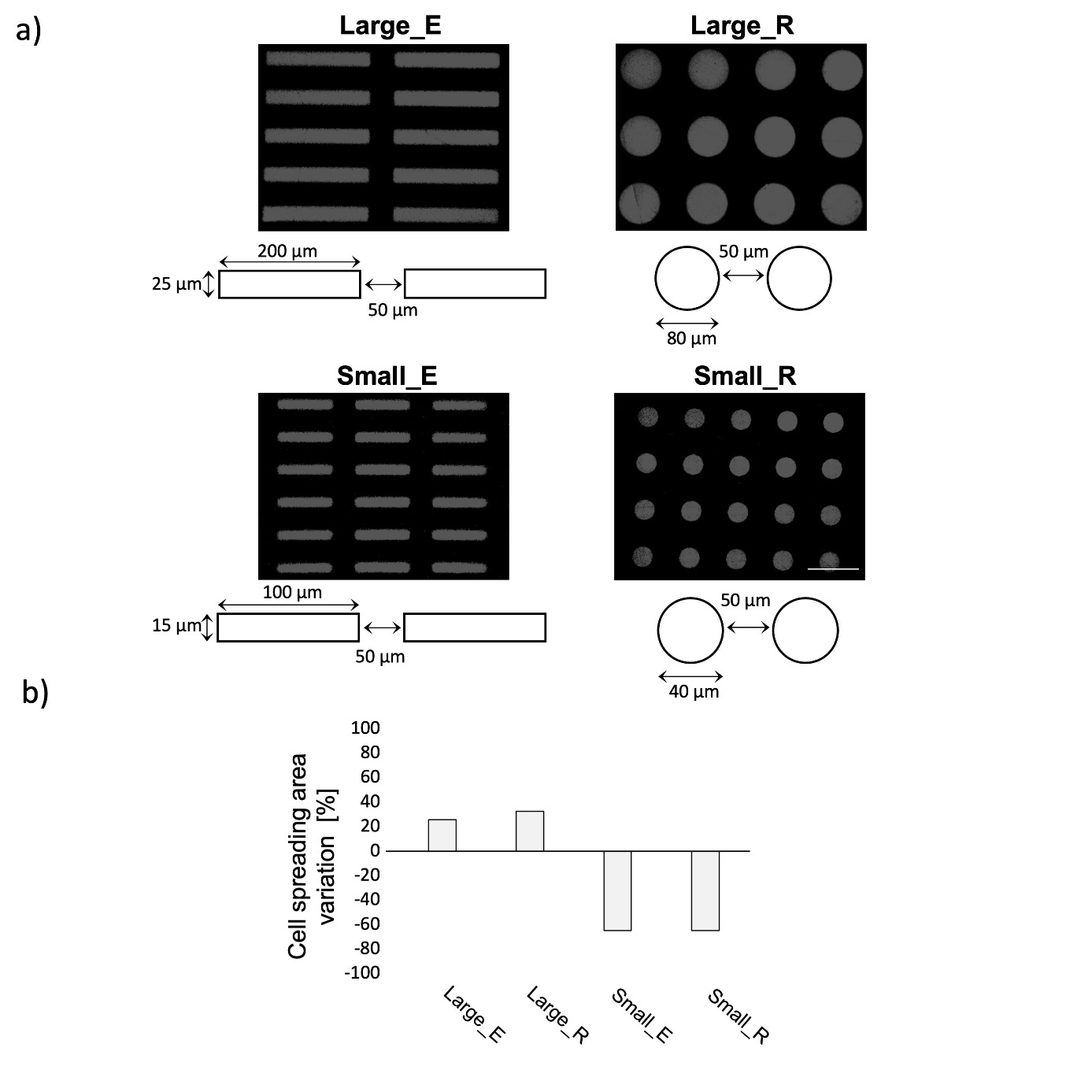


**Figure S1.** a) Features of adhesive islands, Large_E rectangular pattern with length 200µm and width 25µm. Large_R circular pattern with a diameter of 80 µm. Small_E rectangular pattern with length 100µm and width 15µm. Small_R circular pattern with a diameter of 40 µm. b) Cell spreading area changes as function of cell cultured on unpatterned fibronectin coated surface


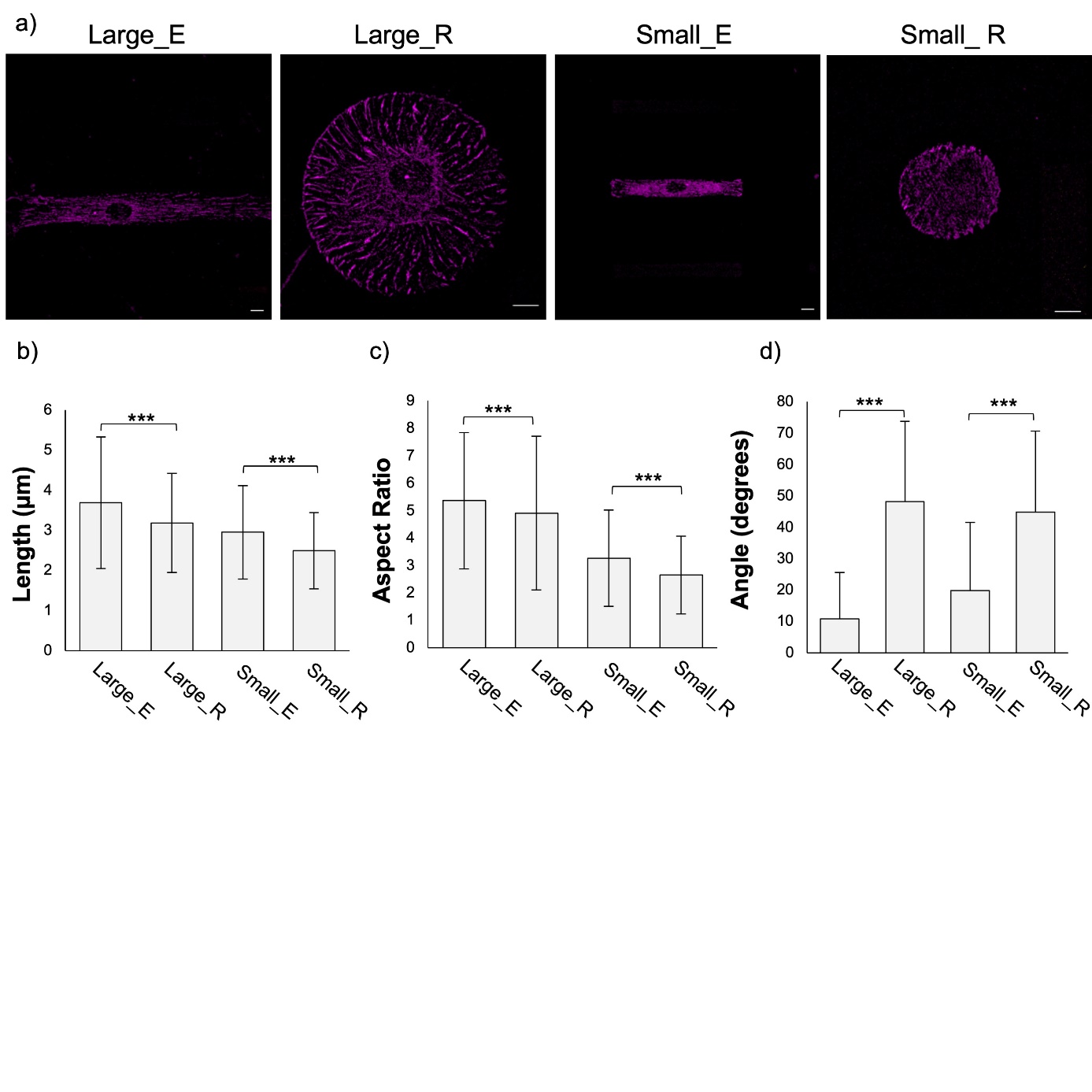


**Figure S2**. a) Confocal images of paxillin (magenta) of ASC cultured on micropatterned surfaces. Scale Bar is 20 µm. Histograms of FA length (b), aspect ratio (c) and orientation (d) of ASCs cultured in all tested conditions. Data are mean ± s.d. Kruskal-Wallis, Post-Hoc Dunn's test (* p < 0.05; ** p < 0.001; ***p < 0.0001).


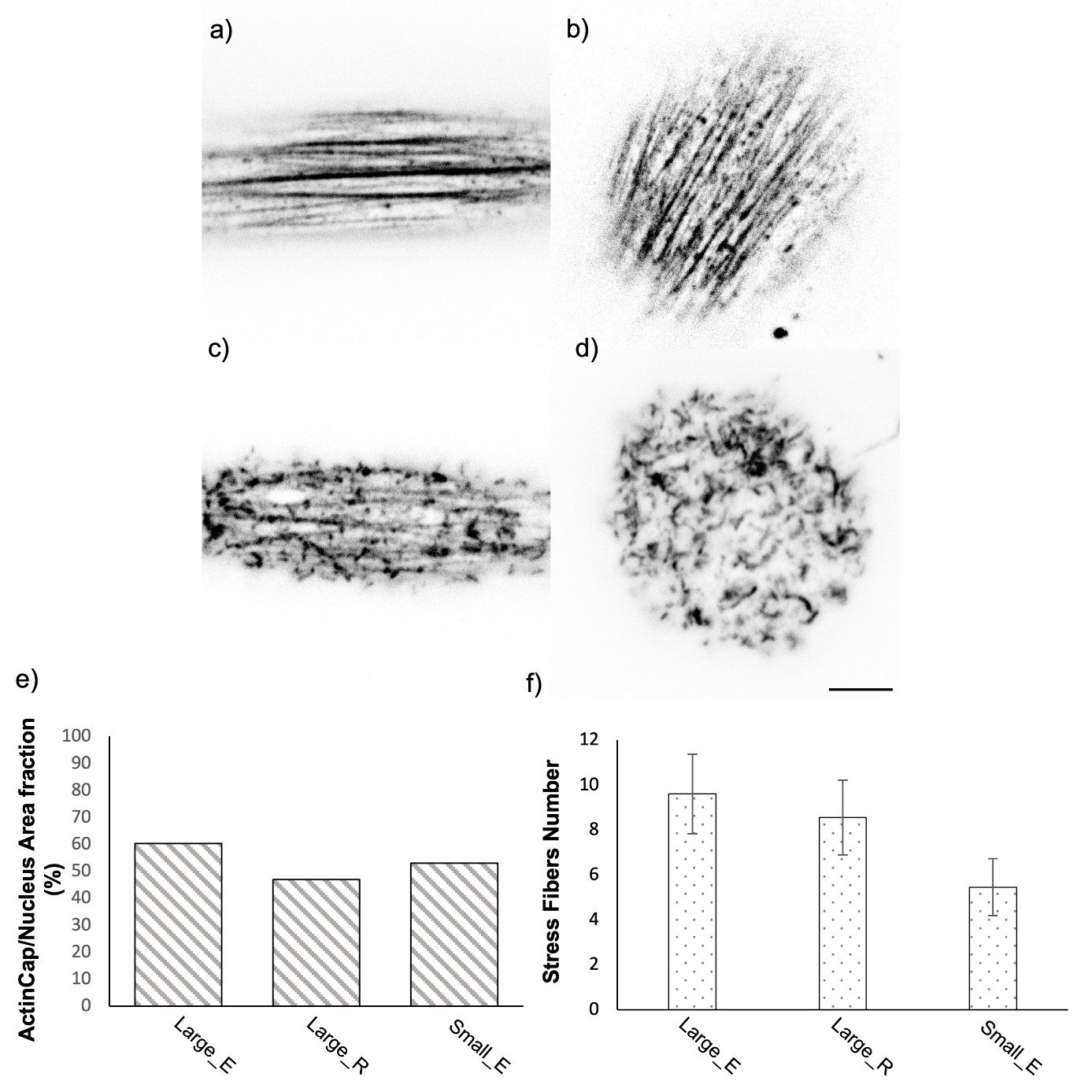


**Figure S3**. Representative Confocal images of ASC actin cap for Large_E (a), Large_R (b), Small_E (c) and Small_R (d). Scale bar is 5 micron. Analysis of perinuclear actin cap area fraction (e) and number of stress fibers (f) in three different micropatterning configurations*.*


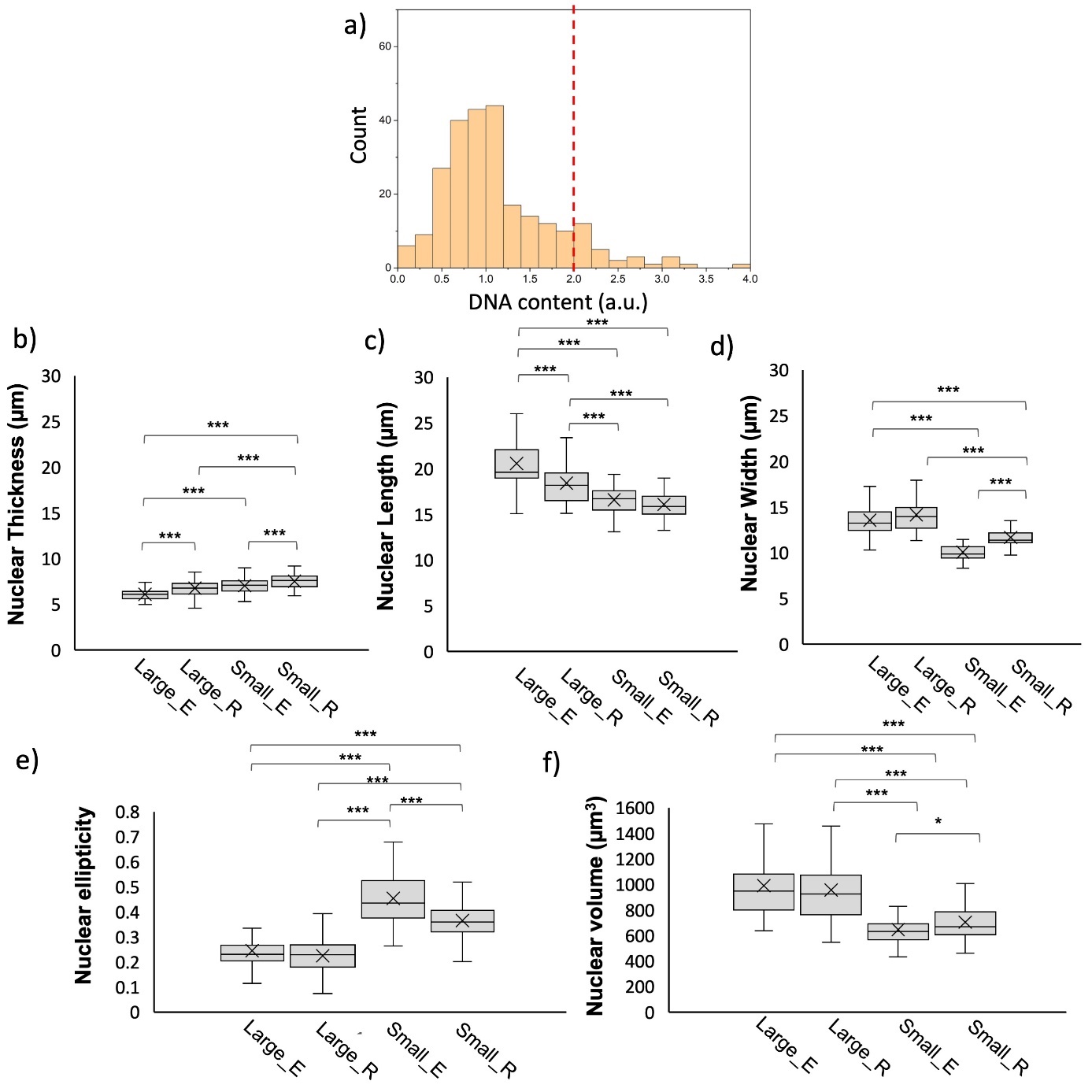


**Figure S4**. a) Histogram of DNA content for the entire cell population as assessed by DAPI intensity measurements. Cells to the left of the red dotted line were considered to be in G1. Box Plots of b) cell nucleus thickness, c) length, d) width, e) ellipticity and f) volume of G1 ASCs cultured on micropatterned surfaces for 24 h. Kruskal-Wallis, Post-Hoc Dunn's test (* p < 0.05; ** p < 0.001; ***p < 0.0001).


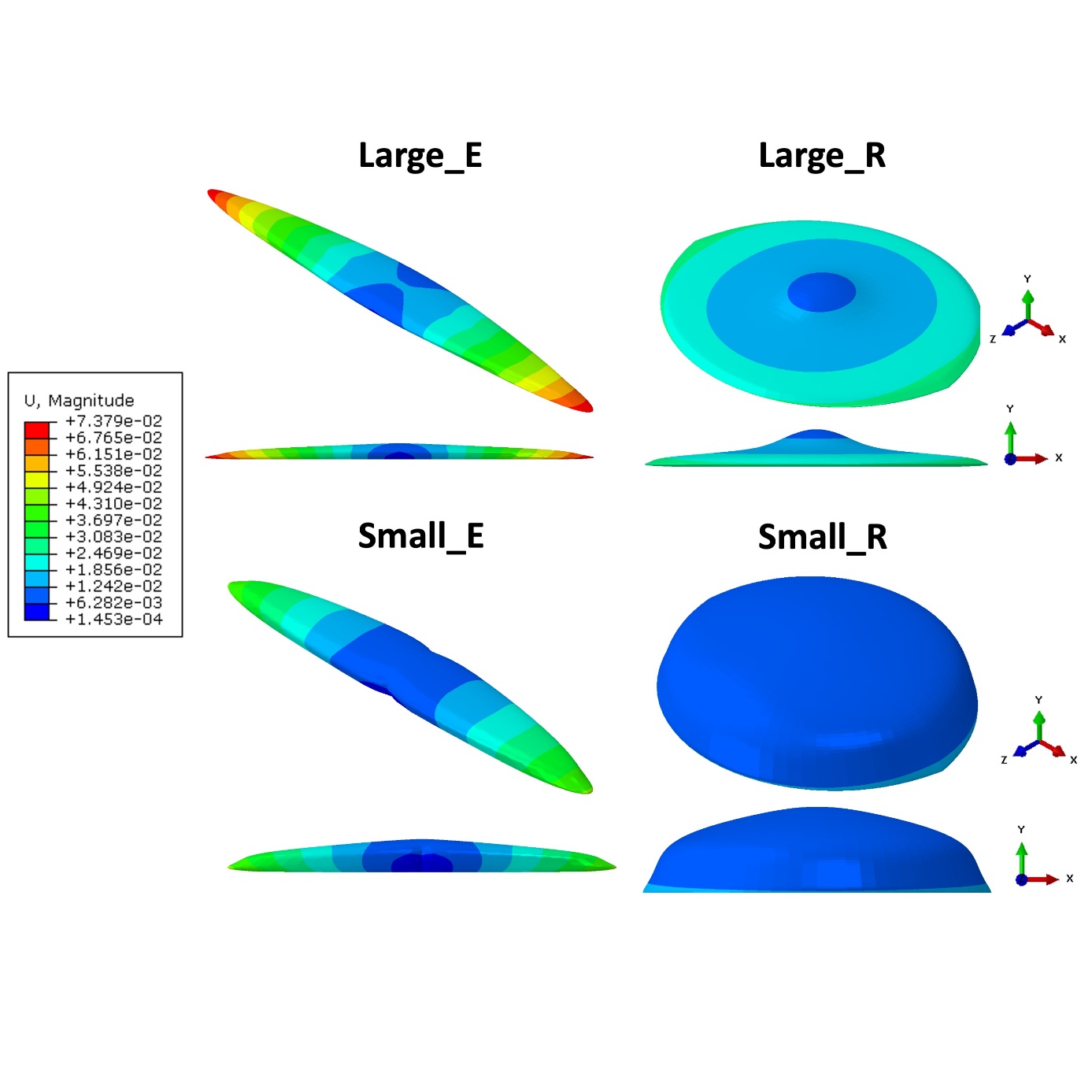


**Figure S5.** Displacement field for all patterns developed on cell surface during the cell spreading.


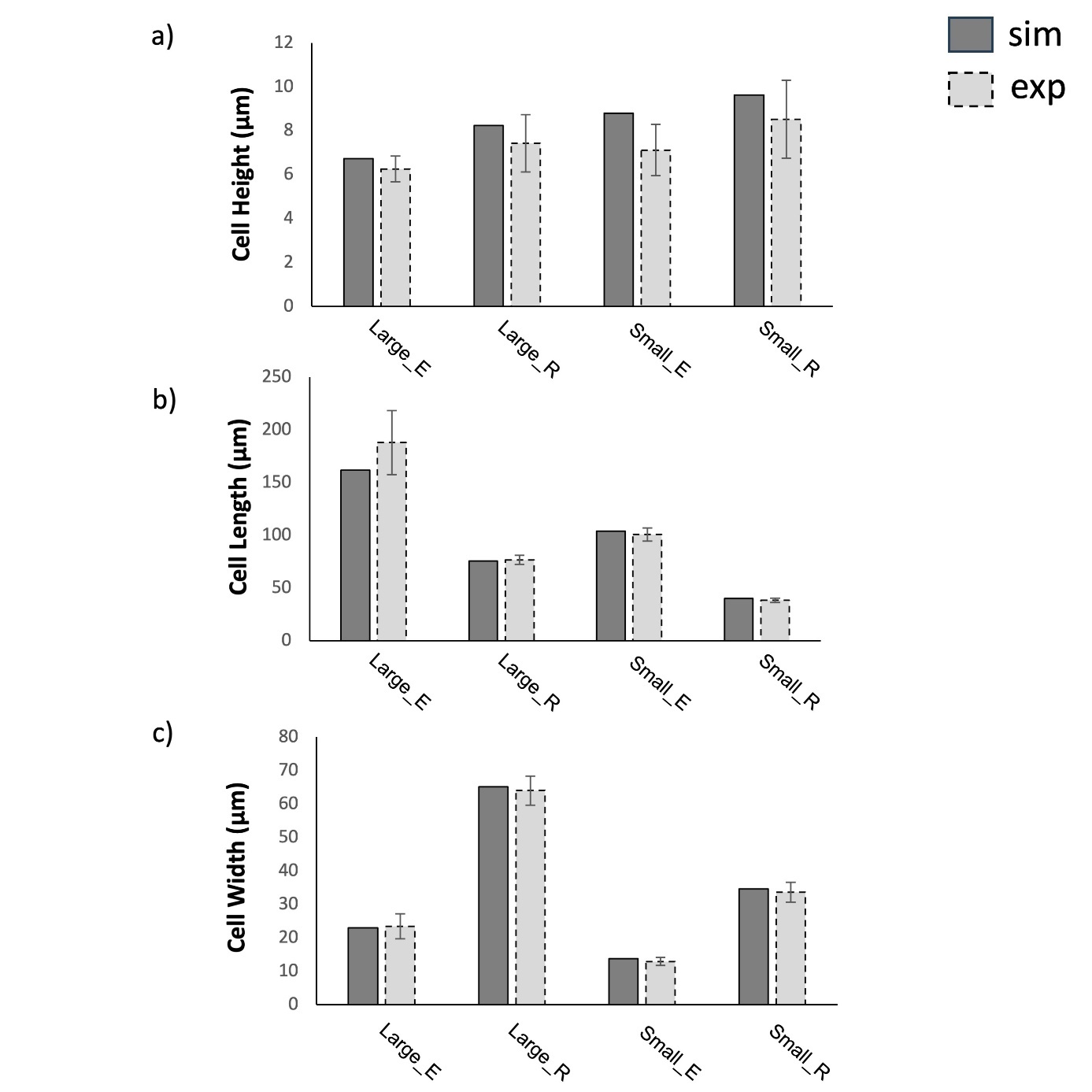


**Figure S6.** Comparison of experimental and simulation results in terms of cell height (a), length (b) and width. The experimental results are expressed as data ± s.d.


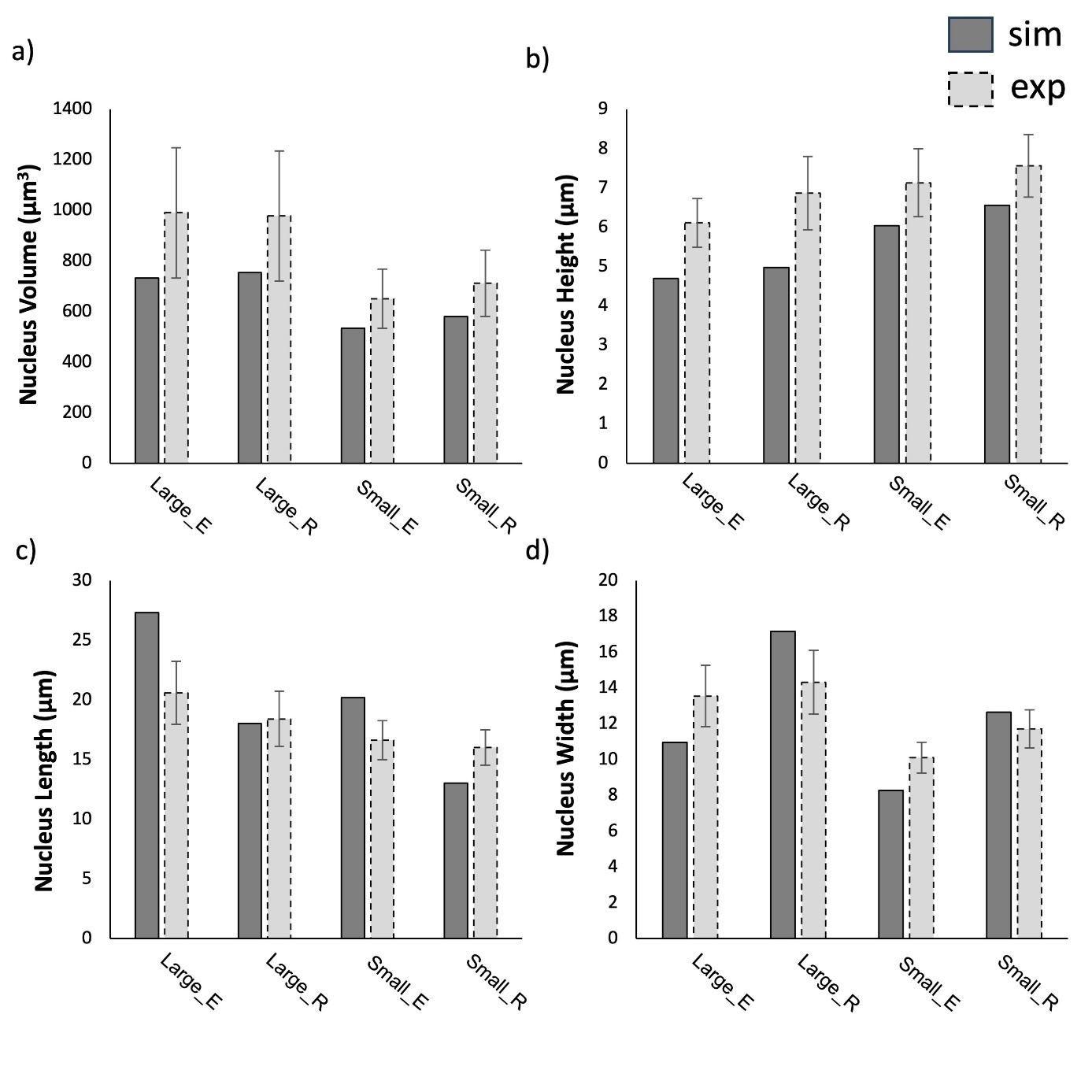


**Figure S7**. Experimental and simulation results in terms of nucleus volume (a), height (b), length (c) and width for all experimental conditions to validate the 3D FEM. The experimental results are expressed as data ± s.d.


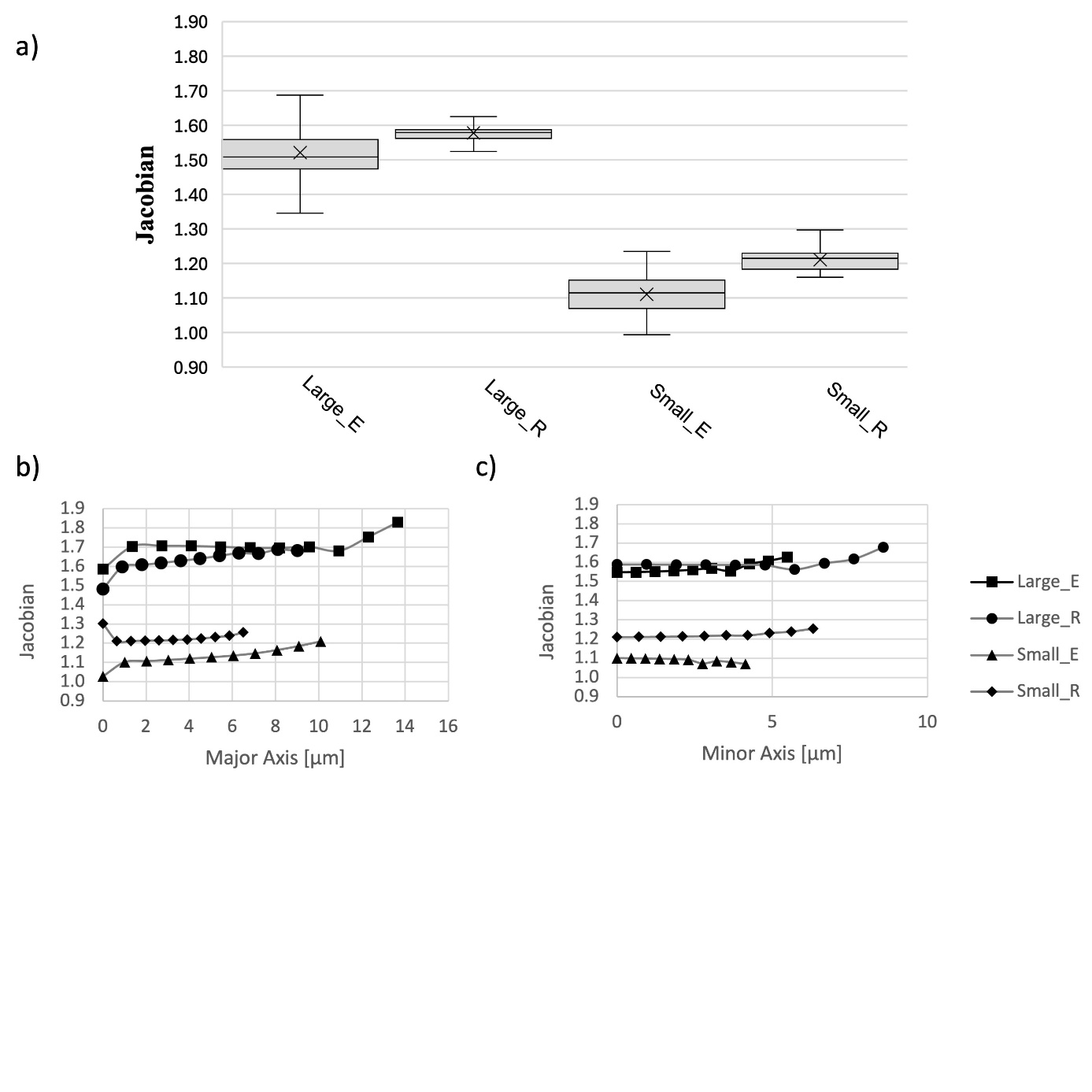


**Figure S8.** a) Box plot of the Jacobian for all nuclear-deformed configurations. b) Jacobian changes of elements along major (b) and minor (c) axis on the nuclear equatorial plane for all micropatterned substrates.


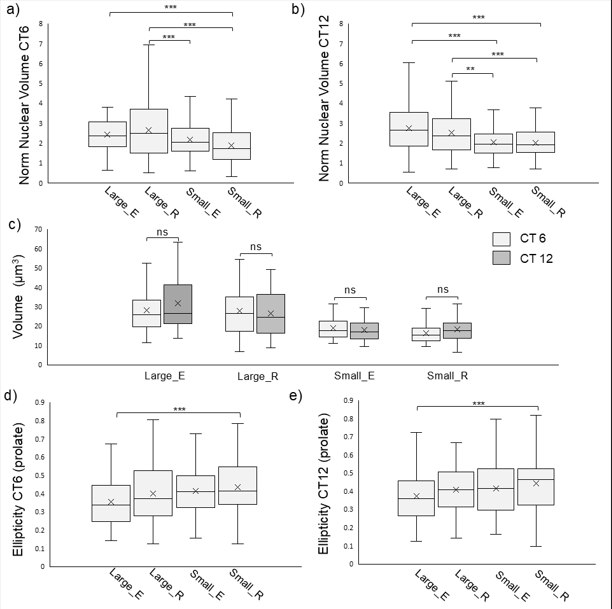


**Figure S9**. Quantification of normalized chromosome volume for CT6 (a) and CT12 (b) for all tested conditions. c) Comparing the volume of CT6 and 12 for each tested condition. d, e) Quantification of CTs ellipticity for CT6 (d) and CT12 (e) for all tested conditions. Kruskal-Wallis, Post-Hoc Dunn's test (* p < 0.05; ** p < 0.001; ***p < 0.0001).


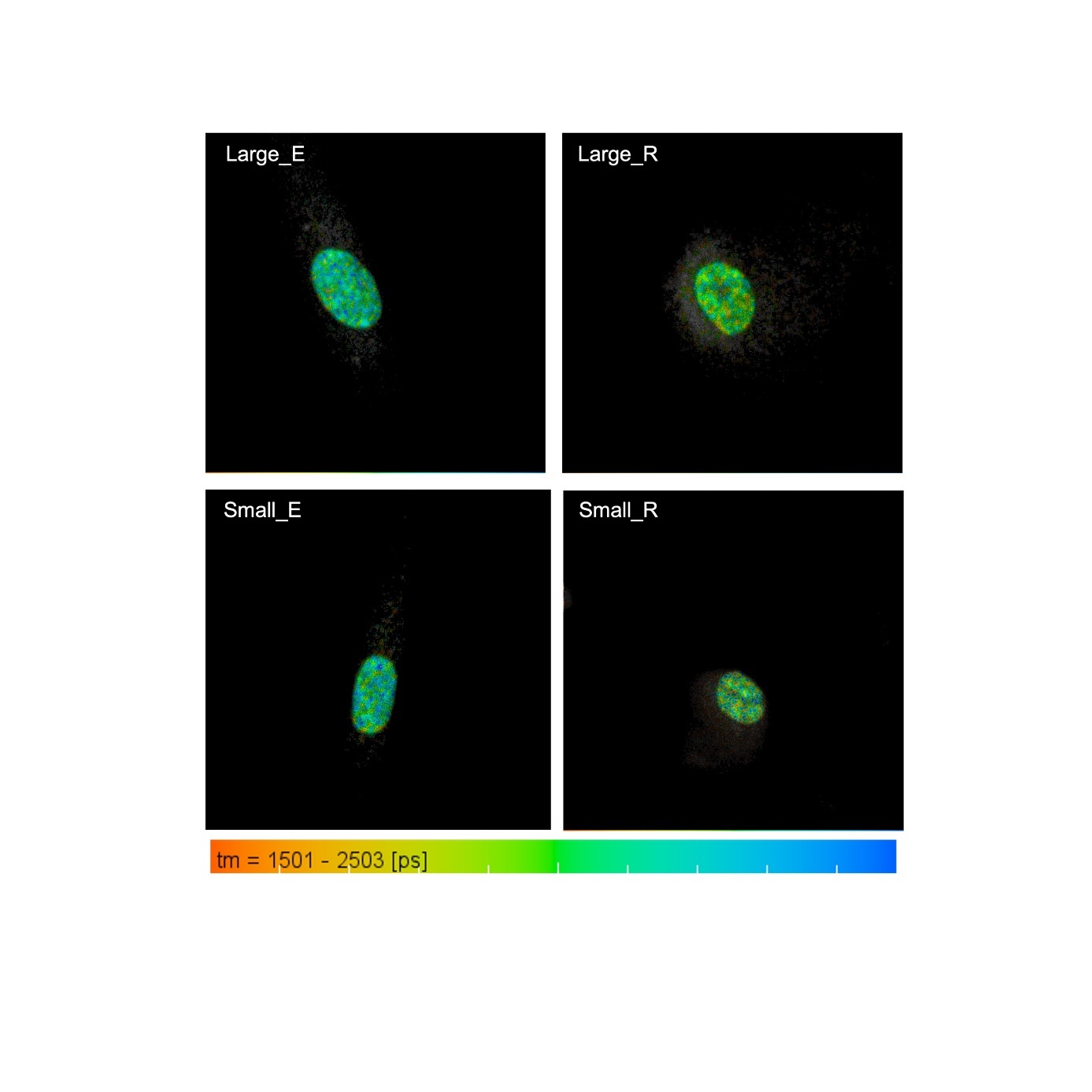


**Figure S10.** FLIM phasor plot analysis of Hoechst fluorescence lifetimes. Nuclear pixels were clustered within defined regions of the phasor plot, allowing visualization and quantification limited to the nuclear area.


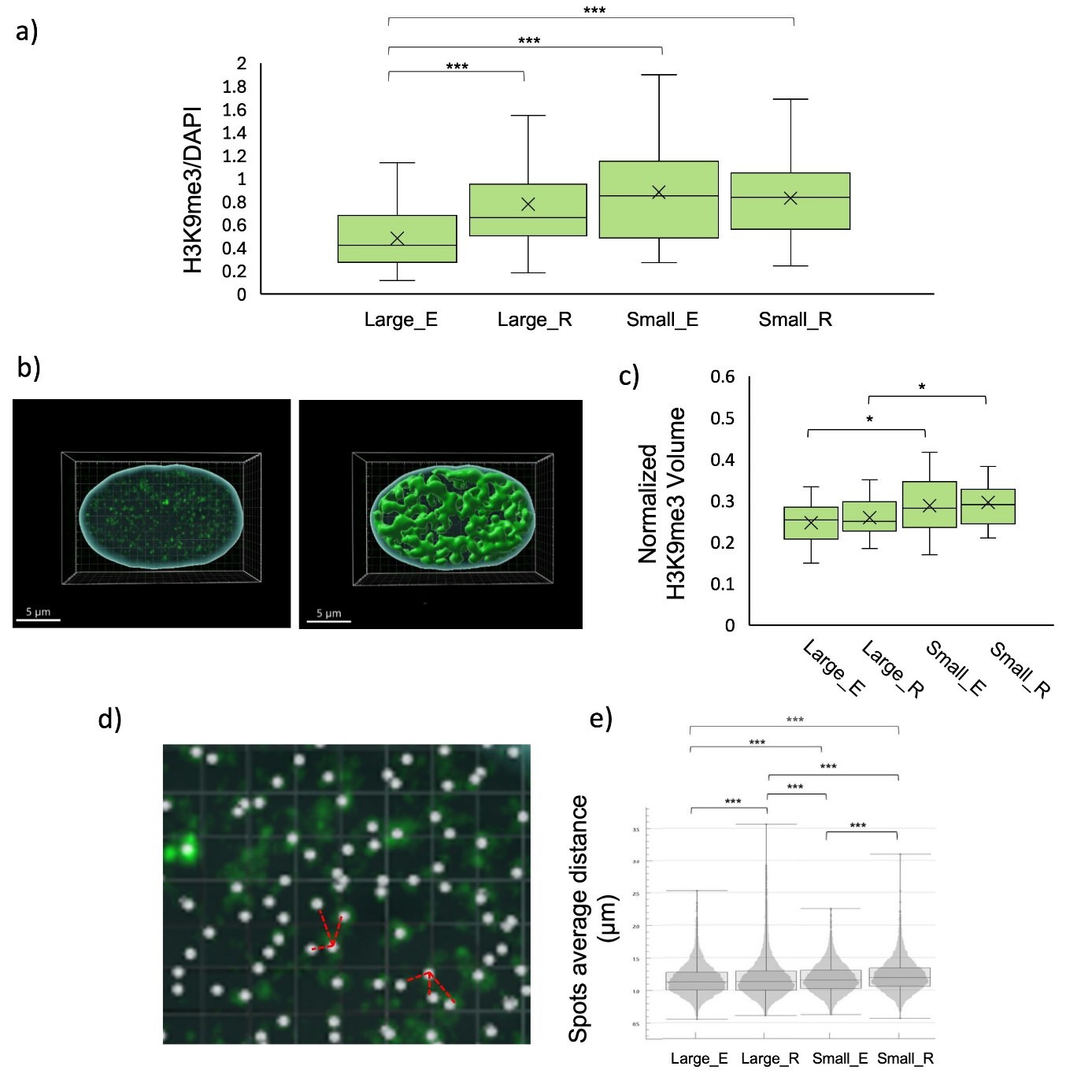


**Figure S11.** a) Quantification of H3K9me3 intensity levels within ASCs nuclei for all tested conditions. The fluorescence intensity of histone modifications was normalized to DAPI. At least 40 nuclei for each condition from three independent experiments were calculated. Kruskal-Wallis, Post-Hoc Dunn's test (* p < 0.05; ** p < 0.001; ***p < 0.0001). b) H3K9me3 volume reconstruction within ASCs nucleus. c) Normalized H3K9me3 volume (V__H3K9me3_ / V__nucleus_) for ASCs cultured on all tested condition. n=40 nuclei for each condition from three independent experiments were calculated d) Representative 3 neighbours (red dotted line) average distance of H3K9me3 foci within cell nucleus e) Quantification of 3 neighbours average distance of H3K9me3 foci within ASCs nuclei for all tested conditions. Kruskal-Wallis, Post-Hoc Dunn's test (* p < 0.05; ** p < 0.001; ***p < 0.0001).


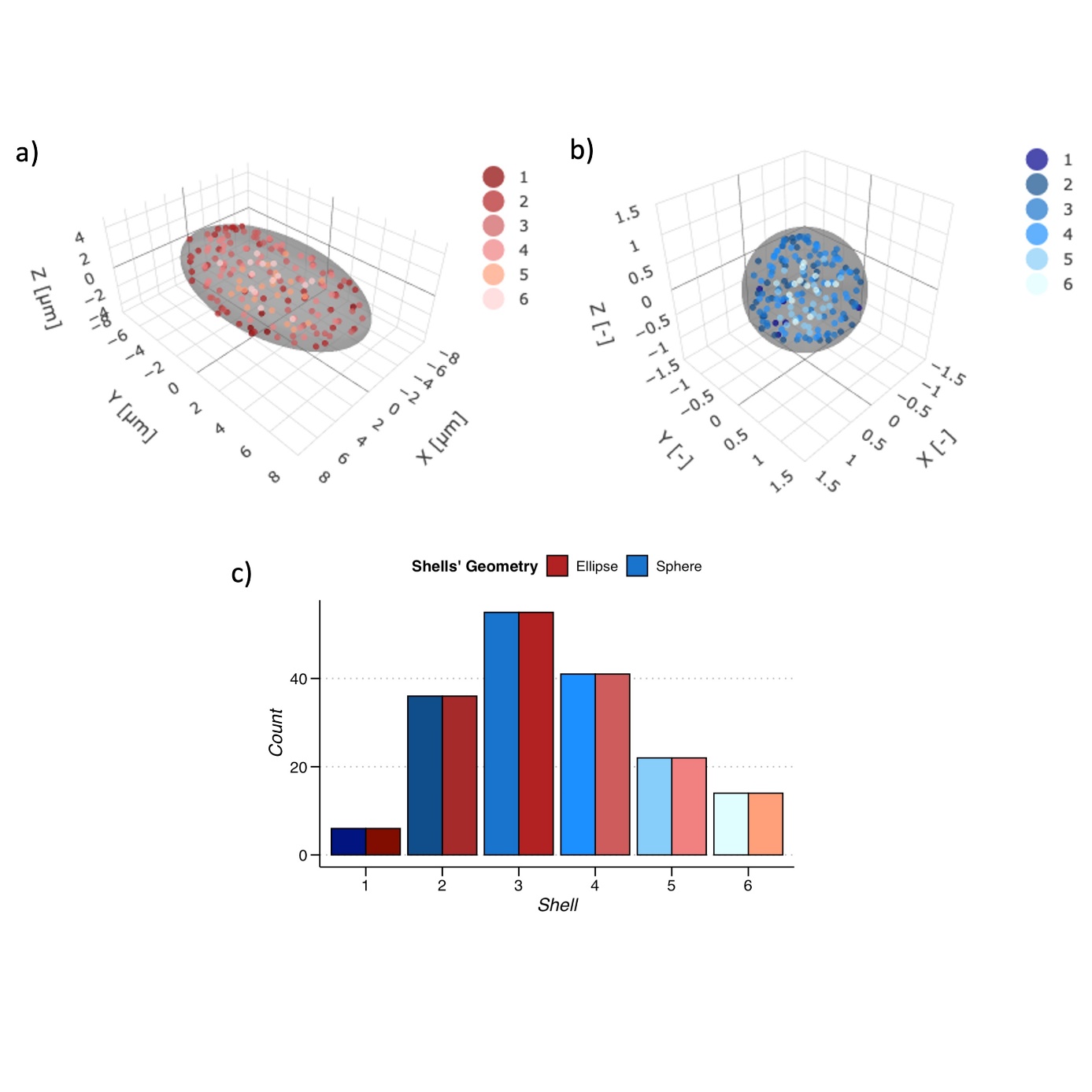


**Figure S12.** Comparison between the classification of heterochromatin foci centroids performed using six iso-volumetric ellipsoidal shells (a) and six iso-volumetric spherical shells after affine transformation (b). Each dot represents the centroid of a heterochromatin focus extracted from Imaris and color-coded according to its shell index, where shell 1 corresponds to the outermost nuclear region and shell 6 to the innermost core. The histogram (c) reports the number of foci assigned to each shell under the two geometries, showing identical distributions. This confirms that the affine mapping preserves spatial relationships and does not introduce bias in the localization of heterochromatin foci.


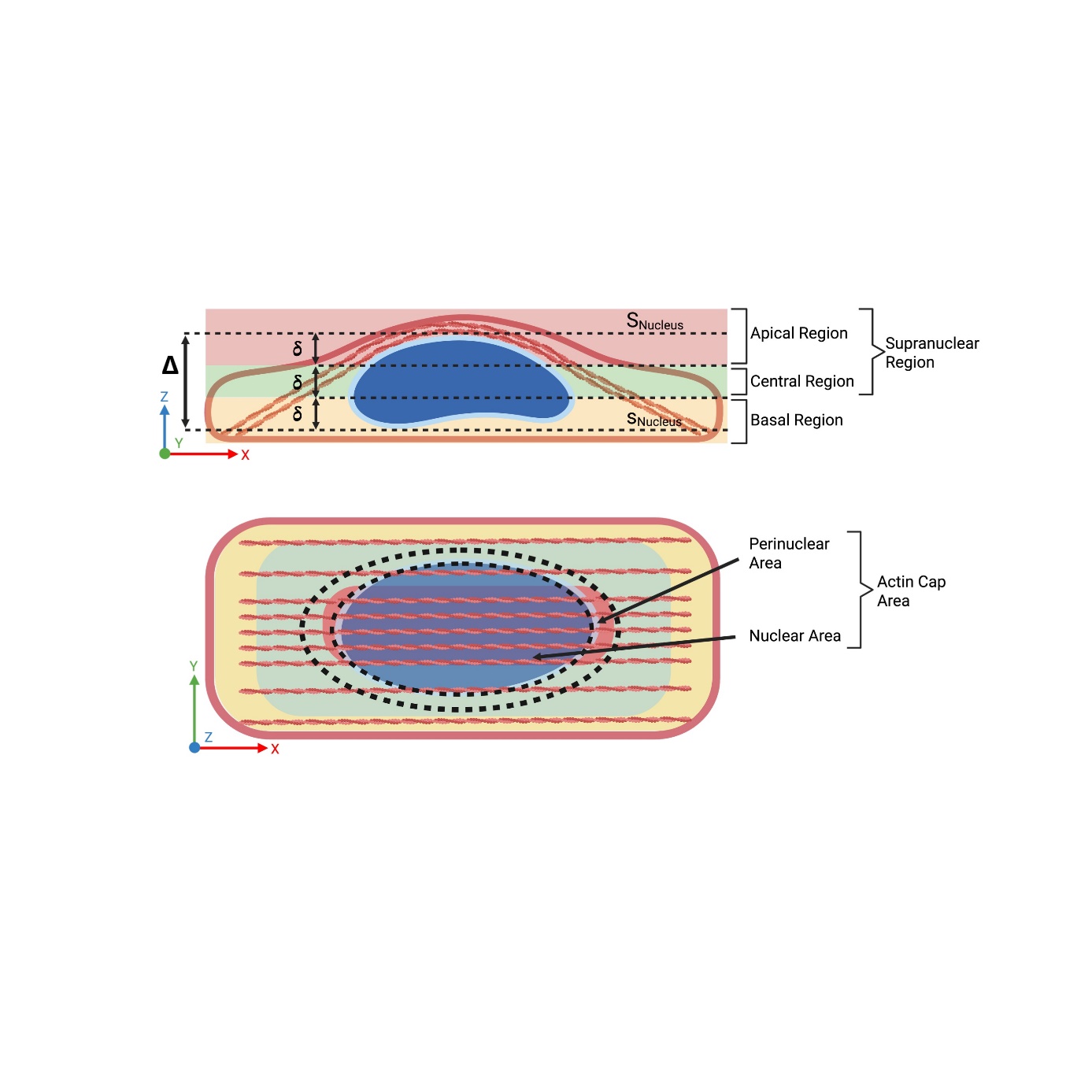


**Figure S13.** Schematic representation of the workflow used to define the supranuclear region and the actin-cap area**.** (Top) The cell volume was conceptually divided into three equal layers along the z-axis using the nucleus as reference. The basal portion below the nucleus was excluded, and the remaining two upper layers—comprising the nucleus and the region above it—were combined and defined as the supranuclear region. (Bottom) Within this supranuclear portion, the actin-cap area was defined by fitting an ellipse to the 2D nuclear projection and expanding its major and minor axes by 25 %, thus including both the nuclear and perinuclear zones where actin-cap fibers are organized.
